# East meets West: current practices and policies in the management of musculoskeletal aging

**DOI:** 10.1007/s40520-019-01282-8

**Published:** 2019-08-02

**Authors:** Weibo Xia, Cyrus Cooper, Mei Li, Ling Xu, Rene Rizzoli, Mei Zhu, Hua Lin, John Beard, Yue Ding, Wei Yu, Etienne Cavalier, Zhenlin Zhang, John A. Kanis, Qun Cheng, Quimei Wang, Jean-Yves Reginster

**Affiliations:** 1 Department of Endocrinology, National Health Commission Key Laboratory of Endocrinology, Peking Union Medical College Hospital, Chinese Academy of Medical Sciences, Beijing, China; 2 MRC Lifecourse Epidemiology Unit, Southampton General Hospital, University of Southampton, Southampton, UK; 3 NIHR Musculoskeletal Biomedical Research Unit, University of Oxford, Oxford, UK; 4 WHO Collaborating Centre for Public Health Aspects of Musculoskeletal Health and Aging, Liege, Belgium; 5 Department of Gynaecology and Obstetrics, Peking Union Medical College Hospital, Chinese Academy of Medical Sciences, Beijing, China; 6 Division of Bone Diseases, Geneva University Hospitals, Faculty of Medicine, Geneva, Switzerland; 7 Department of Endocrinology, Tianjin Medical University General Hospital, Tianjin, China; 8 Department of Orthopaedics, Nanjing Drum Tower Hospital, the Affiliated Hospital of Nanjing University Medical School, Nanjing, China; 9 Department of Aging and Lifecourse, World Health Organization (WHO), 20 Avenue Appia, 1211 Geneva 27, Switzerland; 10 Department of Orthopaedics, Memorial Hospital of Sun Yat-sen University, Guangzhou, China; 11 Department of Radiology, Peking Union Medical College Hospital, Chinese Academy of Medical Sciences, Beijing, China; 12 Department of Clinical Chemistry, University of Liège, CHU Sart Tilman Route 52, Porte 53, Domaine du Sart-Tilman, Liege, Belgium; 13 Department of Osteoporosis and Bone Disease, Shanghai JiaoTong University Affiliated Six People’s Hospital, Shanghai, China; 14 Mary McKillop Health Institute, Australian Catholic University, Melbourne, Australia; 15 Centre for Metabolic Bone Diseases, University of Sheffield Medical School, Sheffield, UK; 16 Department of Osteoporosis and Bone Disease, Huadong Hospital Affiliated to Fudan University, Shanghai, China; 17 Department of Geriatrics, Peking Union Medical College Hospital, Chinese Academy of Medical Sciences, Beijing, China; 18 Division of Public Health, Epidemiology and Health Economics, University of Liège, CHU Sart Tilman B23, 4000 Liege, Belgium; 19 Chair for Biomarkers of Chronic Diseases, Biochemistry Department, College of Science, King Saud University, Riyadh, Kingdom of Saudi Arabia

**Keywords:** Osteoarthritis, Osteoporosis, Sarcopenia, FRAX, Prevention, Treatment

## Abstract

Healthy aging is defined as the process of developing and maintaining the functional ability that enables wellbeing in older age. Healthy aging is dependent upon intrinsic capacity, a composite of physical and mental capacities, and the environment an individual inhabits and their interactions with it. Maintenance of musculoskeletal health during aging is a key determinant of functional ability. Sarcopenia, osteoporosis and osteoarthritis, are a triad of musculoskeletal diseases of aging that are major contributors to the global burden of disease and disability worldwide. The prevention and management of these disorders is of increasing importance with pressure mounting from the aging population. In a new initiative, the Chinese Medical Association, Chinese Society of Osteoporosis and Bone Mineral Research, and the European Society for Clinical and Economic Aspects of Osteoporosis, Osteoarthritis and Musculoskeletal Diseases jointly organized a symposium to discuss current practices and policies in the management of musculoskeletal aging. The meeting allowed experts from Europe and China to share their experience and recommendations for the management of these three major diseases. Discussing and analyzing similarities and differences in their practice should lead, through a mutual enrichment of knowledge, to better management of these diseases, in order to preserve intrinsic capacity and retard the age-related degradation of physical ability. In future, it is hoped that sharing of knowledge and best practice will advance global strategies to reduce the burden of musculoskeletal disease and promote healthy aging tailored to meet the individual patient’s needs.

## Introduction

Healthy aging is defined by the World Health Organization as the process of developing and maintaining the functional ability that enables wellbeing in older age [1]. Healthy aging draws on two main concepts: intrinsic capacity, a composite of all physical and mental capacities that an individual has at any point in time; and the environment they inhabit and their interactions with it. For example, while older people may have limited capacity, they may still be able to shop if they have access to anti-inflammatory medication, an assistive device, and live close to affordable and accessible transport. Central to the concept of healthy aging is an understanding that neither intrinsic capacity nor functional ability remain constant; although both tend to decline with increasing age, life choices or interventions at different points during the life course will determine the trajectory for each individual. Measurement of functional ability and intrinsic capacity may lead to better clinical care, earlier identification of change, identification of early determinants of trajectory, better understanding of population trends, better patient stratification for clinical trials, comparison of the impact of interventions and multiple research outcomes. Maintenance of musculoskeletal health during aging is a key determinant of functional ability.

Typically, decreases in muscle mass and strength, decreases in bone mass and function, and joint tissue degeneration occur with healthy aging. Sarcopenia, a progressive and generalized skeletal muscle disorder leads to diminished physical performance, frailty, and increased risk of falls and fracture in older people with reduced quality of life [2]. Osteoporosis, a skeletal disease of low bone mass and deterioration of bone tissue, increases the risk of bone fragility [3]. Osteoarthritis, a progressive disorder causing joint pain and stiffness, leads to functional decline and loss of quality of life [4, 5]. These musculoskeletal disorders are major contributors to the global burden of disease and disability worldwide [6–8], and prevention and management of these disorders is of increasing importance with pressure mounting from the aging population [9].

In a major new initiative, the Chinese Medical Association (CMA), the Chinese Society of Osteoporosis and Bone Mineral Research (CSOBMR) and the European Society for Clinical and Economic Aspects of Osteoporosis, Osteoarthritis and Musculoskeletal Diseases (ESCEO) jointly organized a 1-day symposium, held in Suzhou, China, on 17th October 2018, to discuss current practices and policies in the management of musculoskeletal aging. The meeting was organized under the auspices of the National Health Commission of the People’s Republic of China, the WHO Collaborating Centre for Public Health Aspects of Musculoskeletal Health and Aging, the Belgian Federal Ministry of Health and Social Affairs and the Belgian Senate. The program identified similarities and differences between approaches in Europe and China for the identification, diagnosis, treatment and management of these three pivotal musculoskeletal disorders. This article summarizes the presentations and discussions shared at the meeting. In future, it is hoped that sharing of knowledge and best practice will advance global strategies to reduce the burden of musculoskeletal disease and promote healthy aging tailored to meet the individual patient’s needs.

## Osteoporosis

Osteoporosis is a systemic skeletal disease characterized by low bone mass and microarchitectural deterioration of bone tissue, with a consequent increase in bone fragility and susceptibility to fracture [10]. The diagnosis of osteoporosis is at the same time an assessment of a risk factor for the outcome of fracture. The remaining lifetime probability of an osteoporotic fracture in postmenopausal women is more than 40% in Western Europe [3]. Common sites for osteoporotic fracture are the spine, hip, distal forearm and proximal humerus, and collectively all osteoporotic fractures accounted for 3.5 million new fragility fractures in men and women in Europe in 2010 [11]. Community-based screening programs for fracture risk are not commonplace but could be effective in reducing hip fractures [12]. Osteoporotic fractures are a major cause of morbidity; hip fractures cause acute pain and loss of function, and result in hospitalization. Vertebral fractures may also cause acute pain and functional loss. The direct cost of osteoporotic fractures was estimated at €37 billion across the 27 EU countries in 2010, which is expected to rise by 25% in 2025 [13].

The operational definition of osteoporosis is based on the *T* score for bone mineral density (BMD) assessed by dual-energy X-ray absorptiometry (DXA) at the femoral neck or spine, and is defined as a value for BMD 2.5 standard deviations (SD) or more below the young female adult mean [14]. Several clinical factors contribute significantly to fracture risk over and above that provided by BMD measurement including: age, sex, low body mass index (BMI), previous fragility fracture, parental history of hip fracture, glucocorticoid treatment, current smoking, alcohol intake of three or more units daily and causes of secondary osteoporosis [15, 16], and are incorporated into the Fracture Risk Assessment tool (FRAX^®^). Country-specific FRAX should be used to assess fracture probability in postmenopausal women; there are 63 FRAX models available covering 80% of the world population, used in 173 countries, and incorporated into 130 guidelines worldwide, including those of the ESCEO and the International Osteoporosis Foundation (IOF) [15], and those of the CMA [17].

### European practice for fracture risk assessment

**Fig. 1:**
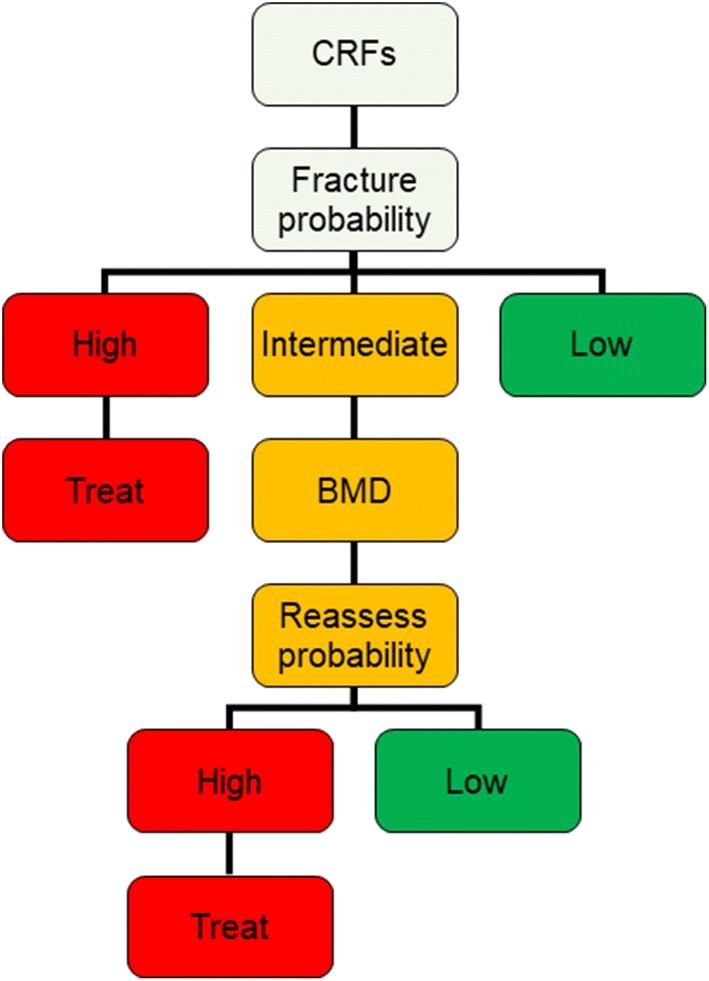
Case-finding strategy for osteoporosis from ESCEO-IOF guidance [15]. *BMD* bone mineral density, *CRF* clinical risk factor. Reprinted from: Kanis JA et al. [15] with permission from Springer 2019

The ESCEO-IOF guidance for the diagnosis and management of osteoporosis in postmenopausal women outlines a case-finding strategy based upon clinical risk factor identification and stratification into high, intermediate and low fracture risk (Fig. 1) [15]. A prior fragility fracture is a sufficient signal that treatment of osteoporosis may be recommended. The intervention threshold in women without a prior fracture can be set at the age-specific 10-year fracture probability derived from FRAX that is equivalent to women with a prior fragility fracture [15]. The availability of BMD measurement using DXA varies by country [10]. Thus, with limited access to DXA, BMD measurement may be reserved for those individuals who lie close to the intervention threshold, which may reduce the need for DXA by up to 60% [15].

### Osteoporosis in China

The size of the aging population is growing rapidly in China. For Chinese women, the prevalence of osteoporosis measured at at least one site increases with age from 24% in those aged 50–59 years to 83% in those aged over 80 years [18]. The prevalence of osteoporosis at the lateral spine region in this population was 9–11% at the age of 40–49 and 37–41% at the age of 50–59, and 0.5–4% at the age of 40–49 and 4–22% at the age of 50–59 years at other skeletal sites [18]. The risk of vertebral fracture increases among postmenopausal Chinese women from 13% at age 50–59 years to over 50% after age 80 years [19]. Studies suggest that the rate of hip fractures is increasing in areas of Asia that are undergoing urbanization [20]. Data collected between 1990–1992 and 2002–2006 in Beijing, China, showed a 2.76-fold (95% confidence interval [CI] 2.68, 2.84) increase in age-specific hip fracture risk among women aged > 50 years and 1.61-fold (95% CI 1.56, 1.66) increase in men from 1990 to 2006 [20]. Osteoporosis-related fractures cause a substantial economic burden [21], which is predicted to increase markedly over the coming decades. Around 2.3 million osteoporotic fractures occurred in China in 2010 among people aged ≥ 50 years, costing US$9.5 billion; both the number and costs of osteoporosis-related fractures are estimated to double by 2035, reaching 6 million fractures costing US$25.4 billion by 2050 [22].

### Case-finding strategy for osteoporosis management in China

**Fig. 2:**
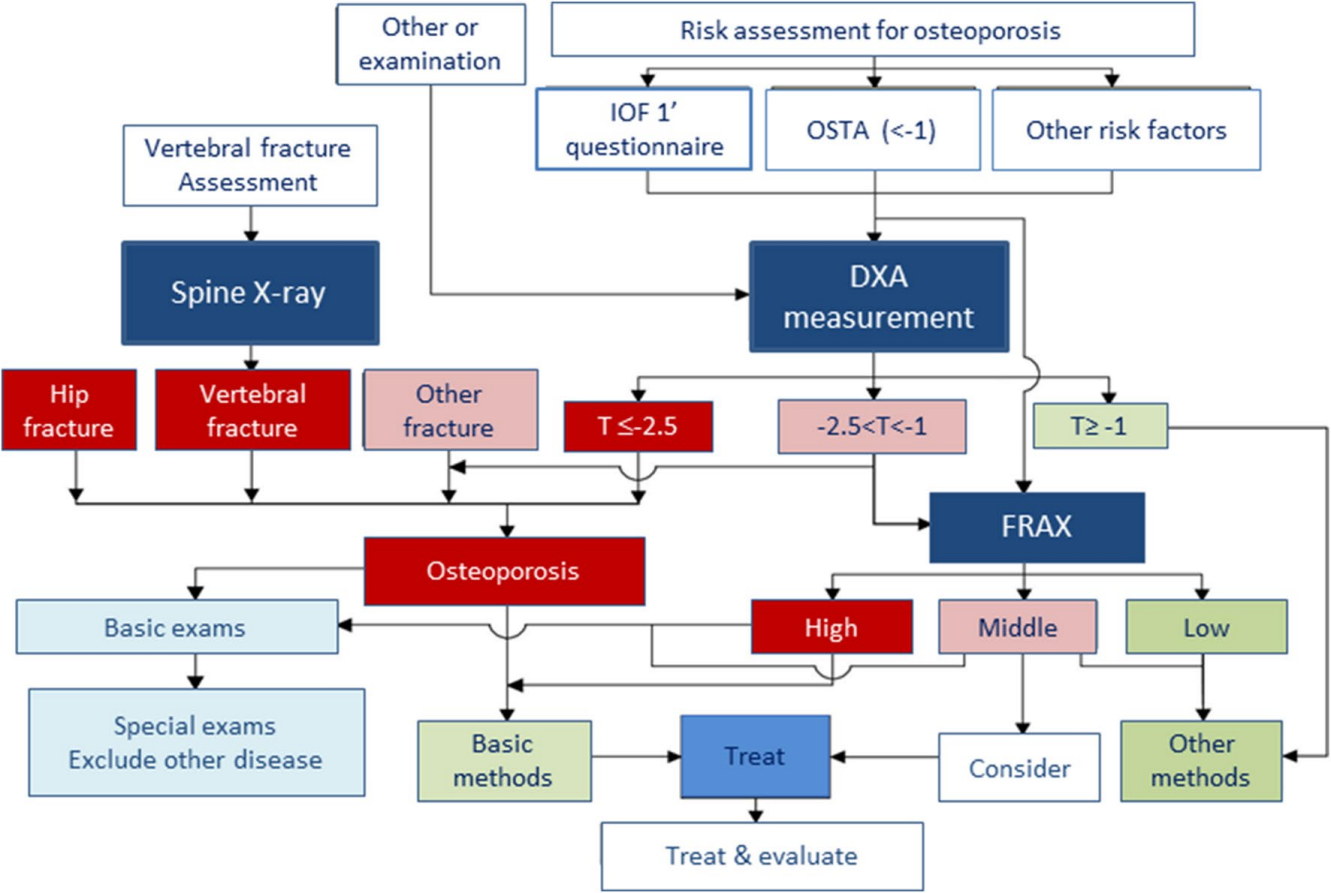
Case-finding strategy for osteoporosis from Chinese Medical Association guidance. Adapted from CSOBMR [17]. *DXA* dual-energy X-ray absorptiometry, *FRAX* fracture risk assessment tool, *IOF* International Osteoporosis Foundation, *OSTA* Osteoporosis Self-assessment Tool for Asians

In order to better identify those at risk of osteoporotic fractures, and prevent these from occurring, the CMA has outlined a case-finding strategy in its guidelines for primary osteoporosis [17]. Risk assessment may be performed using the IOF primary questionnaire for risk of osteoporosis, the Osteoporosis Self-assessment Tool for Asians (OSTA), or other risk factors, and be performed after a fall or during screening. Case diagnosis may involve X-ray to determine presence of fracture and DXA to measure BMD (Fig. 2). FRAX assessment is confined to patients with low bone mass (osteopenia) and thereafter stratified into high, middle and low fracture risk [17].

In China, there is often a gap between patients and doctors in terms of osteoporosis care: patients do not know where to find a doctor who can treat osteoporosis, and doctors do not know where to find patients without a fracture but at high risk of osteoporosis. One way to bridge the gap may be through the development of new Osteoporosis Centers in hospitals. Win Over Osteoporosis (WOO) is a national project of China, focused on enhancing the education of osteoporosis and screening for high-risk osteoporosis. A second option may be to optimize a tiered medical diagnosis and treatment system, through case referrals from primary to middle and tertiary care. Another route is to promote Healthcare Consortiums such as the National Osteoporosis Alliance Hierarchical Healthcare System (NOAH). Lastly, knowledge dissemination may be achieved through education of family doctors and general practitioners and patient education and screening in the community.

### European practice for using BTMs in the diagnosis and management of osteoporosis

Bone remodeling is a tightly coupled process of bone resorption and bone formation that occurs to preserve the mechanical integrity of bone and regulate calcium homeostasis [23]. Bone turnover biomarkers (BTMs) are enzymes of osteoblasts and osteoclasts or by-products of bone remodeling, and are categorized into bone formation and bone resorption markers. BTMs may identify changes in bone remodeling within a relatively short time interval before changes in BMD can be detected. Thus, BTMs may be used to predict bone loss in pre- and post-menopausal women and to monitor the efficacy of treatment in osteoporotic women. Research evidence suggests that BTMs may provide information on fracture risk independently from BMD, so that fracture risk prediction might be enhanced by their inclusion in assessment algorithms [24]. The IOF and International Federation of Clinical Chemistry and Laboratory Medicines (IFCC) recommend that a marker of bone formation, serum procollagen type I N propeptide (PINP), and a marker of bone resorption, serum C-terminal telopeptide of type I collagen (CTX-I), are used as reference markers of bone turnover and for prediction of fracture risk and monitoring of osteoporosis treatment [25].

Although there are significant associations between BTMs and incident fracture risk, the association is modest and BTMs are not commonly used for the prediction of fracture risk [26]. However, BTMs are generally used for the follow-up of osteoporosis treatment efficacy, as there are significant positive associations between the reduction in BTM and the reduction in fracture risk which support the use of BTM in monitoring treatment [25]. In Europe, BTMs are recommended for the follow-up of patients treated with anti-osteoporotic drugs because of poor adherence [27]. Adherence to oral bisphosphonates is as low as 50% at 1 year; low adherence significantly jeopardizes the anti-fracture efficacy and cost-effectiveness of treatment.

Bone turnover biomarkers concentration can be affected by different conditions that are important to take into consideration when interpreting results; this includes intra- and inter-subject variability, different measurement methods, variability in different diseases, and between different agents [25, 28–30]. Guidelines from The National Bone Health Alliance (NBHA) recommend standardized sample handling and patient preparation for CTX-I and PINP measurements to reduce pre-analytical variability [31].

### BTMs in the management of osteoporosis in China

The proper application and assessment of BTMs in clinical practice is important. To achieve this aim, the Japan Osteoporosis Society and the CSOBMR have developed guidelines for the use of BTMs in osteoporosis [32–34]. The normal range of BTMs in Chinese people has been established through the Chinese Bone Turnover Marker study [35, 36]; the study found no significant differences in CTX-I and PINP concentrations among different cities with different latitudes. Serum osteocalcin (OC) and bone-specific alkaline phosphatase (BAP) are found to be the key determining factors of early BMD decreases in Chinese middle-aged women [37].

Data linking BTMs with fracture risk are lacking within Asian populations; from the Singapore Chinese Health Study, a population-based prospective cohort of Chinese men and women (45–74 years), higher serum levels of BTMs, namely PINP, OC, N-terminal crosslinking telopeptide of type I collagen (NTX-I) and CTX-I, were associated with increased risk of hip fracture [38]. Among Chinese postmenopausal women in the Peking Vertebral Fracture study, serum CTX-I and PINP levels negatively correlated with BMD, and CTX-I was higher in postmenopausal women with sustained fracture or vertebral fracture [39]. Further prospective longitudinal studies are needed to explore the predictive role of BTMs in relation to bone loss and fracture risk in the Chinese population.

The clinical value of BTMs in other diseases also needs to be evaluated. Suppressed BTMs are associated with increased osteoporotic fracture risk in non-obese postmenopausal Chinese women with type 2 diabetes mellitus [40], while subcutaneous fat area correlates with OC in postmenopausal women and is negatively associated with BMD [41]. The effect of treatment on BTMs and BMD has been evaluated in Chinese subjects with osteoporosis; for example, zoledronic acid (IV 5 mg/year) increased BMD at the lumbar spine over 2 years and rapidly decreased BTMs (PINP and CTX-I) [42], and treatment with low-dose alendronate (70 mg every 2 weeks) in women with postmenopausal osteopenia/osteoporosis increased lumbar spine and hip BMD and decreased serum BTMs [43]. A systematic review of teriparatide (SC 20 μg/day) for treatment of Asian patients with osteoporosis at high risk for fracture, shows a sustained increase in bone formation markers (PINP, BAP) and bone resorption markers (CTX-I, NTX-I) [44]. Novel BTMs, such as osteoprotegerin, receptor activator of nuclear factor-κB ligand, and sclerostin have emerged and are worthy of further research [45, 46].

### European guidelines for management of osteoporosis

**Fig. 3:**
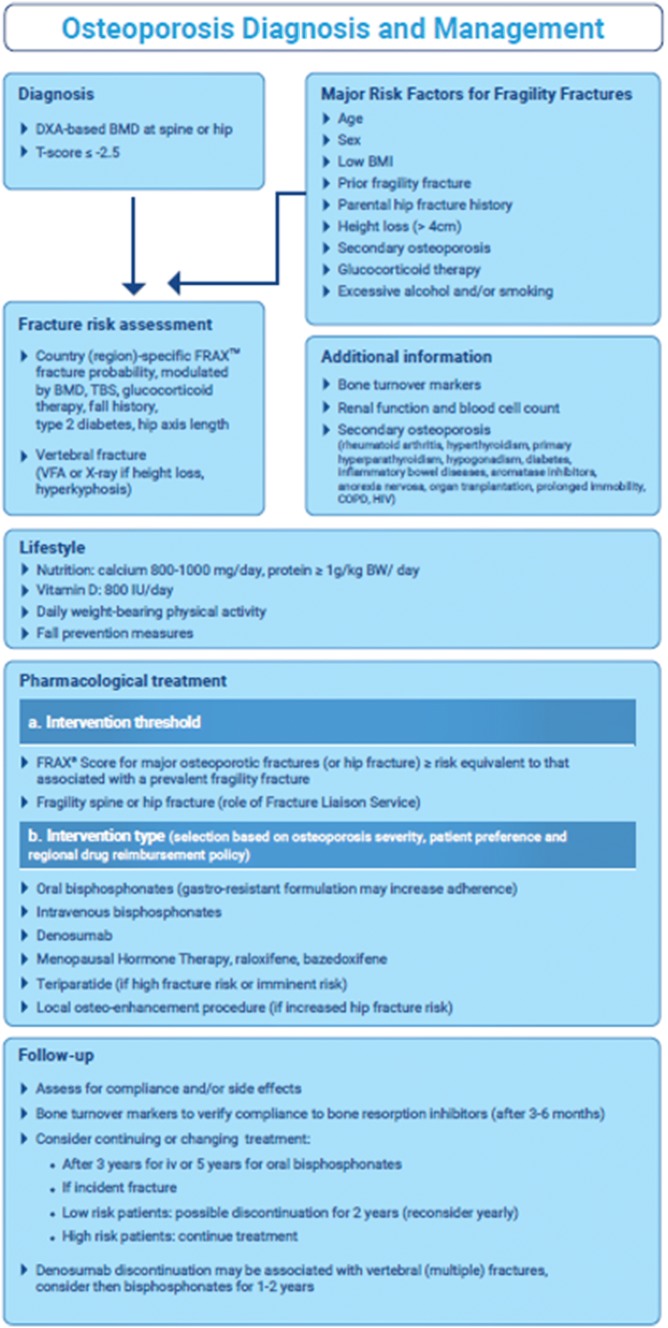
ESCEO-IOF algorithm for diagnosis and management of osteoporosis in postmenopausal women [47]. *BMD* bone mineral density, *BMI* body mass index, *COPD* chronic obstructive pulmonary disease, *DXA* dual-energy X-ray absorptiometry, *FRAX* fracture risk assessment tool, *HIV* human immunodeficiency virus, *TBS* trabecular bone score. Reprinted from: Kanis JA et al. [47] with permission from Springer 2019

The 2019 update of the European guidance for the diagnosis and management of osteoporosis in postmenopausal women contains new information on the general and pharmacological management of osteoporosis (Fig. 3) [47], including data on the long-term effects of dietary intake and on fracture risk with drug treatment discontinuation [15].

Age-dependent intervention thresholds provide guidance for decision-making based upon the 10-year probability of major osteoporotic and hip fracture derived from FRAX [48]. Women aged over 65 years with a prior fracture can be considered for treatment without further intervention, while BMD measurement may be more appropriate in younger postmenopausal women [15].

#### Lifestyle and dietary measures

European recommendations include a daily calcium intake of 800–1200 mg and sufficient dietary protein, ideally achieved through dairy products. A daily dose of 800 IU cholecalciferol is advised for postmenopausal women at increased risk of fracture. Calcium supplementation is appropriate if the dietary intake is below 800 mg/day, and vitamin D supplementation considered in patients at risk of, or showing evidence of, vitamin D insufficiency [15].

Regular weight-bearing exercise is advised, tailored to the needs and abilities of the individual patient. A history of falls should be obtained in individuals at increased risk of fracture with further assessment and appropriate measures undertaken in those at increased risk [15].

#### Pharmacological intervention

The oral bisphosphonates (alendronate, risedronate and ibandronate) are recommended as initial treatment in the majority of cases. Gastro-resistant formulations of oral bisphosphonates may increase adherence, and thus may be a preferred option [47]. Treatments should be reviewed after 3–5 years treatment with bisphosphonate. In women intolerant to oral bisphosphonates (or in those for whom they are contraindicated), intravenous bisphosphonates or denosumab provides the most appropriate alternatives, with raloxifene, or menopausal hormone therapy as additional options [15].

Teriparatide is preferentially recommended for patients at high risk of fracture. Fracture risk should be reassessed after a new fracture, regardless of when it occurs. The risk of new clinical and vertebral fractures increases in patients who stop treatment. Withdrawal of denosumab therapy is associated with a rebound in vertebral fracture rate; bisphosphonate therapy can be considered after discontinuation of denosumab [15]. Local osteo-enhancement procedures may be considered if there is an increased risk of hip fracture [47].

There is little evidence to guide decision-making beyond 10 years of treatment and management options in such patients should be considered on an individual basis.

### Osteoporosis treatment under Chinese guidelines

Osteoporotic fractures cause considerable mortality and morbidity in the aging population. The current diagnosis and medical treatment following fragility fractures is still insufficient in mainland China and needs to be optimized [49]. A Chinese nationwide survey identified that greater awareness of guidelines for osteoporosis management by orthopedists is needed to enable them to better manage their patients. For example, a significant number had prescribed doses of vitamin D and calcium lower than the recommended levels [50].

Global mapping has identified a prevalence of low average dietary calcium intake in Asian countries (< 400 mg/day) and low vitamin D status (25–49 nmol/L) [51, 52]. Chinese guidelines recommend calcium intake of 1000–1200 mg/day and vitamin D of 800–1200 IU daily for older adults [34]. Active vitamin D (alfacalcidol or calcitriol) are recommended for osteoporotic patients who are elderly, have renal dysfunction, or have 1α-hydroxylase deficiency.

There is evidence that calcitriol may reduce BMD loss and improve quality of life in the short-term among elderly Chinese patients with osteoporosis, although further research is needed to assess long-term effects. Calcitriol in combination with other anti-osteoporotic agents can improve BMD, BTMs, bone pain and reduce the risk of new vertebral fracture [53].

Alendronate in combination with vitamin D_3_ shows greater increases in BMD at the lumbar spine and greater reduction in BTMs compared with calcitriol in Chinese postmenopausal women [54]. The combination could provide further benefit to the vitamin D status of postmenopausal patients with vitamin D insufficiency/deficiency [55]. Higher baseline PINP and CTX-I, and greater PINP and CTX-I decreases at 6 months were associated with greater improvement in lumbar spine BMD after alendronate plus vitamin D3 treatment [56].

## Osteoarthritis

Osteoarthritis (OA) is a degenerative disease with joint pain as the main symptom accompanied by functional limitation, with morning stiffness common in knee and hip joints and joint swelling common in hand OA. Hip and knee OA together are the eleventh highest contributor to global disability; the years of life lived with OA-related disability was estimated at 17 million in 2010 [7]. OA is a progressive disorder that requires long-term management with various treatment options over the course of the disease. Treatment guidelines, including those from the ESCEO and CMA, exist to assist physicians in the diagnosis and treatment of OA [57–59]. The CMA guidelines include diagnostic criteria for hip, knee and hand OA (Table 1) [59]. While the pathogenesis of OA remains unclear, there are no anti-OA drugs that can effectively halt disease progression, and eventually treatment with arthroplasty surgery may be necessary. How to delay the development of OA, improve quality of life of patients with OA, and even how to prevent OA, are questions that are still to be answered.

**Table 1:** Guidelines from the Chinese Medical Association for the diagnosis of osteoarthritis of the hip, knee and hand. Table compiled from Xing-Ming et al. [59]

Osteoarthritis diagnostic criteria	Hip	Knee	Hand: interphalangeal joint
1	Repeated hip pain in the last month	Repeated knee pain in the last month	Pain, sore, stiffness of interphalangeal joint
2	Erythrocyte sedimentation rate ≤ 20 mm/h	X-ray film shows the typical manifestations of osteoarthritis	The number of joints with bony enlargement in ten interphalangeal joints ≥ 2
3	X-ray shows osteophyte formation	Age ≥ 50 years	The number of distal interphalangeal joints with bone enlargement ≥ 2
4	X-ray shows narrowing of joint space	Morning stiffness ≤ 30 min	The number of metacarpophalangeal swellings < 3
5	–	Bone friction sound during activity	The number of deformed joints in ten interphalangeal joints ≥ 1
Diagnostic conditions	1 + 2+3 or 1 + 3+4	1 + (any 2 symptoms in 2, 3, 4, 5)	1 + (any 2 symptoms in 2, 3, 4, 5)

### European management of knee OA

**Fig. 4:**
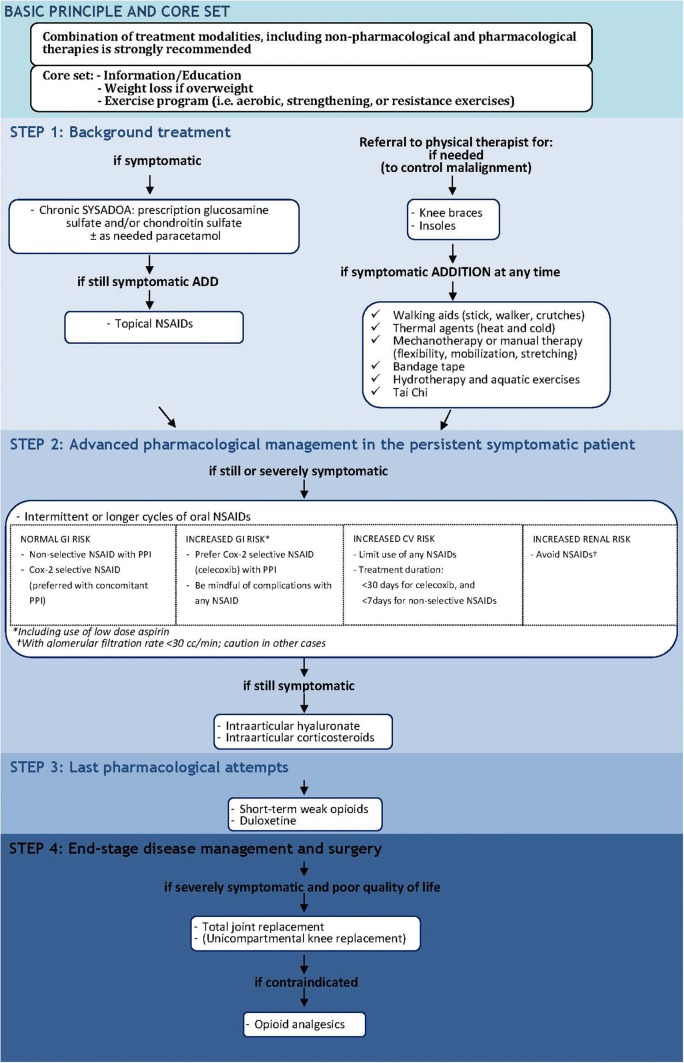
ESCEO updated algorithm for management of knee osteoarthritis [58]. *COX-2* cyclooxygenase-2, *CS* chondroitin sulfate, *CV* cardiovascular, *GI* gastrointestinal, *GS* glucosamine sulfate, *IA* intra-articular, *NSAID* non-steroidal anti-inflammatory drug, *PPI* proton pump inhibitor, *SYSADOA* symptomatic slow-acting drugs in osteoarthritis, *OA* osteoarthritis. Reprinted from: Bruyere O et al. [58] with permission from Elsevier 2019

In 2014, the ESCEO published recommendations for the management of knee OA in the form of a treatment algorithm that prioritized interventions through progressive steps [57]. The algorithm was well-received internationally and translated and published in Chinese [60]. Since 2014, considerable new evidence has emerged leading the ESCEO to publish an updated algorithm (Fig. 4) [58]. The 2019 algorithm retains a core set of education, weight loss as appropriate, and exercise alongside a combination of non-pharmacological and pharmacological treatments. In step 1, background pharmacological therapy is recommended with symptomatic slow-acting drugs in osteoarthritis (SYSADOAs). There are many different agents in the class of SYSADOAs including glucosamine, chondroitin, diacerein, and avocado soybean unsaponifiables (ASU), which are supported by variable degrees of clinical efficacy data. Thus, among all SYSADOAs the ESCEO strongly recommends specifically the use of pharmaceutical-grade prescription glucosamine sulfate (pCGS) and chondroitin sulfate, for which the evidence base is unequivocal and there are no safety issues [57, 58, 61, 62]. Due to only weak efficacy and increasing safety concerns, paracetamol (acetaminophen) is only recommended for short-term analgesia at doses no greater than 3 g/day. Topical non-steroidal inflammatory drugs (NSAIDs) may be added in step 1 for cyclic add-on analgesia, for which moderate efficacy is demonstrated with good safety profile [63–65]. Oral NSAIDs are recommended as step 2 advanced pharmacological treatment, as intermittent or cyclical treatment to control pain rather than chronic use due to a risk of gastrointestinal (GI) and cardiovascular (CV) adverse events (AEs) with all oral NSAIDs [66, 67]. In the case of contraindication to NSAIDs, or if the patient is severely symptomatic, intra-articular (IA) treatment may be considered. IA hyaluronic acid (HA) is an effective treatment with beneficial effects on pain, function and patient global assessment [68–72]. IAHA has a slow onset of action, with superior longer-lasting efficacy compared with IA corticosteroids [73]. IA corticosteroids have shown efficacy in the short-term (2–4 weeks) and may be used to treat knee effusion; no benefit of repeat courses of IA corticosteroids on symptoms and joint structure modification in the long term (2 years) [74]. Step 3, last pharmacological options before recourse to surgical intervention for severely symptomatic patients are short-term weak opioids for which there is good evidence of analgesic benefit in knee OA albeit with a high degree of AEs of the GI, dermatological and central nervous systems [75–77].

### Safety of analgesic agents in the treatment of OA

Recent reports of safety issues associated with paracetamol have raised concerns over its routine chronic use, due to increasing evidence of GI, CV, hepatic and renal AEs, especially at the upper end of standard analgesic doses (up to 4 g/day) [78–80]. A dose–response effect was reported, with increased odds of reduced renal function (odds ratio [OR] 1.80; 95% CI 1.02, 3.18) [81, 82]. An increase in upper GI AEs (ulcers, hemorrhages) was found with relative rate elevation to 1.49 (95% CI 1.34, 1.66) [83], and an increased risk for all CV AEs was reported with increased risk ranging from 1.19 (95% CI 0.81, 1.75) (dose 325–650 mg/week) to 1.68 (95% CI 1.10, 2.57) (> 4875 mg/week) [84]. Further, an increase in mortality risk was associated with paracetamol use versus non-use of 1.63 (95% CI 1.58, 1.68) [83, 85].

Recent meta-analyses of the safety of NSAIDs suggest that all non-selective NSAIDs and cyclooxygenase-2 (COX-2) inhibitors have the potential for GI and CV toxicity [67, 86]. A meta-analysis of 280 trials of NSAIDs versus placebo (*N* = 124,513) and 474 trials of one NSAID versus another NSAID (*N* = 229,296) found that all NSAID regimens increase upper GI complications compared with placebo (COX-2 inhibitors rate ratio [RR] 1.81; 95% CI 1.17, 2.81; diclofenac RR 1.89; 95% CI 1.16, 3.09; ibuprofen RR 3.97; 95% CI 2.22, 7.10; and naproxen RR 4.22; 95% CI 2.71, 6.56) [86]. The risk of upper GI AEs with non-selective NSAIDs is attenuated by use of gastroprotectant proton pump inhibitors (PPIs) [87].

The absolute risk of myocardial infarction (MI) associated with NSAID use is estimated at around 0.5–1% per year [88]. While it was previously thought that selectivity of the NSAID for the COX-2 enzyme governed the CV toxicity profile, recent results suggest that CV risk may be drug specific; rofecoxib is associated with an increased risk of CV events, while celecoxib is associated with a lower incidence of MI (OR 0.58; 95% CI 0.40, 0.86) and stroke (OR 0.60; 95% CI 0.41, 0.89) [89], and celecoxib is non-inferior to naproxen or ibuprofen for CV safety [90]. However, a safety meta-analysis of COX-2 inhibitors (celecoxib, rofecoxib, etoricoxib, and valdecoxib) found a significant increase in CV AEs, even when rofecoxib was excluded, specifically hypertension, congestive heart failure and peripheral and generalized edema [66], which is consistent with other findings [91].

### Prevention and treatment of OA in China

There is a high burden from knee OA among the Chinese population, as identified in the China Health and Retirement Longitudinal Study [92]. Among 17,459 subjects (52.1% women; mean age 59.1 years) included in this analysis, the prevalence knee of OA was 8.1%, higher in women (10.3%) than men (5.7%), and with significant geographical differences: highest in the Southwest (13.7%) and Northwest (10.8%), and lowest in North China (5.4%) and Eastern Coastal (5.5%). Years lived with disability (YLD) for knee OA in China was estimated at 4,149,628, and YLD rate (per 100,000 population) was 968. Female sex, older age, rurality, lower education, and lower gross domestic production per capita were associated with high YLD rates for knee OA. Among urban populations the prevalence of hip OA is around 1%, and for hand OA the prevalence is 3% in men and 5.8% in women [59].

With the aging of China’s population, the incidence of OA is gradually rising and research interest in OA in China is steadily increasing [93]. Great efforts have been made in the prevention and treatment of OA in China, and updated guidelines for the diagnosis and treatment of OA in China were issued in 2018 [59]. These guidelines, updated from the earlier 2007 edition, have added diagnostic criteria for interphalangeal joint OA (Table 1), revised diagnostic criteria for knee OA, and introduced Kellgren and Lawrence (KL) classification according to radiograph, and Outerbridge grading of OA (classification according to articular cartilage injury).

The overall treatment principle is a ladder of escalating treatment and individualized treatment according to the patient’s age, gender, weight, self-risk factors, lesion location and severity. Basic treatment includes health education, physiotherapy, and exercise therapy [94]. Recommended pharmacological treatment includes the use of topical or oral NSAIDs [95], intra-articular injection of hyaluronic acid (HA) [96] or glucocorticoid, selective use of SYSADOAs, and cautious use of paracetamol and opioids [59].

Platelet-rich plasma (PRP) is an autologous and multifunctional platelet concentrate of the blood that stimulates the cartilage healing process and improves the damage caused by articular disease and is under investigation for the treatment of OA. PRP treatment is significantly more effective than HA treatment in reducing pain measured on the Western Ontario and McMaster Universities Arthritis Index (WOMAC) pain score (*p* < 0.05) and International Knee Documentation Committee (IKDC) subjective score [97, 98]. Further, PRP and HA in combination significantly improved arthralgia, reduced humoral and cellular immune responses and promoted angiogenesis, which improved the patients’ histological parameters compared with PRP or HA treatment alone.

The effectiveness of traditional Chinese medicine treatment has been explored in OA, using an artificial tiger bone powder. After 8 weeks of treatment, the combination of Chinese traditional medicine and NSAID resulted in a significantly greater change of Lequesne index and improvement of clinical symptoms compared with either treatment alone (*p* < 0.05) [99].

In terms of restorative treatment, improvements in surgical methods, such as arthroscopy and osteotomy and arthroplasty have been achieved [100, 101]. Arthroscopy is effective in the treatment of knee OA with mechanical symptoms and can relieve symptoms for up to 2 years without elevating the risk of arthroplasty [102]. Around 400,000 patients have undergone arthroplasty annually in China since 2014. Unicompartmental knee arthroplasty can result in sustained improvement in pain and functional scores lasting for 6–8 years [103], and delivers similar results to total knee arthroplasty but with the advantage of less trauma, and faster recovery [100].

In future, new treatments for OA may be derived from developments in basic research, which is unravelling the etiology of OA; research on the role of transforming growth factor-beta [104], H-type vessels [105], and metalloproteinase degradation [106] is ongoing.

### Imaging of OA

Radiography is the gold standard imaging technique used in clinical practice for evaluation of known or suspected OA and in research to visualize signs of OA including joint space narrowing (cartilage thickness and meniscal integrity), osteophytes, subchondral sclerosis and cysts. Radiographic outcome measures are currently the only approved endpoints in clinical trials. Semiquantitative assessments using radiography include the KL grading system and the Osteoarthritis Research Society International (OARSI) atlas, which is more sensitive to longitudinal radiographic changes than KL grading [107]. The ability of magnetic resonance imaging (MRI) to visualize all joint structures in three dimensions has deepened understanding of the natural history of OA. MRI can provide both semiquantitative and quantitative measurement of OA; semiquantitative including cartilage, meniscus ligaments, bone marrow lesions (BMLs), synovium, joint effusion, cyst and loose bodies with a grading system, and quantitative measurement of cartilage thickness and volume, BMLs, meniscus and joint effusion using specialized software and readers. MRI protocols exist that provide imaging data on multiple articular structures and features relevant to knee OA to support a broad range of measurement methods while balancing requirements for high image quality and consistency against practical considerations [107, 108]. A number of scoring systems have been developed for semiquantitative assessment using MRI that can be applied to different joints including: the Whole Organ Magnetic Resonance Imaging Score (WORMS), the Kornaat Osteoarthritis Scoring System (KOSS), the Boston–Leeds Osteoarthritis Knee Score (BLOKS), the MRI Osteoarthritis Knee Score (MOAKS), the Hip Osteoarthritis MRI Score (HOAMS), and the Oslo Hand Osteoarthritis MRI score (OHOA-MRI) [109]. Other techniques including sonography and positron emission tomography–computed tomography (PET–CT) may be used; PET–CT has high sensitivity but low specificity and poor spacial resolution [109].

## Sarcopenia

Sarcopenia is a disease of aging, characterized by progressive and generalized loss of skeletal muscle strength and low muscle mass or quality leading to low physical performance and a risk of adverse outcomes such as physical disability, falls, poor quality of life and death [110, 111]. Muscle mass is measured using body imaging techniques, such as DXA and bioimpedance analysis (BIA), muscle strength using the handgrip test, and physical performance using gait speed or the Short Physical Performance Battery (SPPB) [111, 112].

### Sarcopenia identification in Europe

Progress in the identification and management of sarcopenia has been hampered by a lack of clear consensus on the definition and diagnosis of sarcopenia, as several definitions have emerged from various groups using different assessment tools and cut-off values including: the European Working Group on Sarcopenia in Older People (EWGSOP) [110]; the International Sarcopenia Consensus Conference Working Group (ISCCWG) [113]; the Foundation for the National Institutes of Health (FNIH) [114]; the Society of Sarcopenia, Cachexia and Wasting Disorders [115]; and the Asian Working Group for Sarcopenia (AWGS) [112]. Using these five screening methods, the prevalence of sarcopenia was determined to range from 5.7 to 16.7% [116]. The most specific tool was found to be the EWGSOP algorithm (up to 91%), which also has high validity for predicting the rate of falls [117]. All the screening tools performed well in identifying subjects who do not suffer from sarcopenia and who should not, with certainty, benefit from further assessment of muscle mass. However, all the screening tools had low sensitivity, and thus do not have the necessary performance for efficient screening of the general population [116]. In the same population, the SarQoL^®^ was able to discriminate sarcopenic from non-sarcopenic subjects with regard to their quality of life, regardless of the definition used for diagnosis as long as the definition includes an assessment of both muscle mass and muscle function [118].

**Fig. 5:**
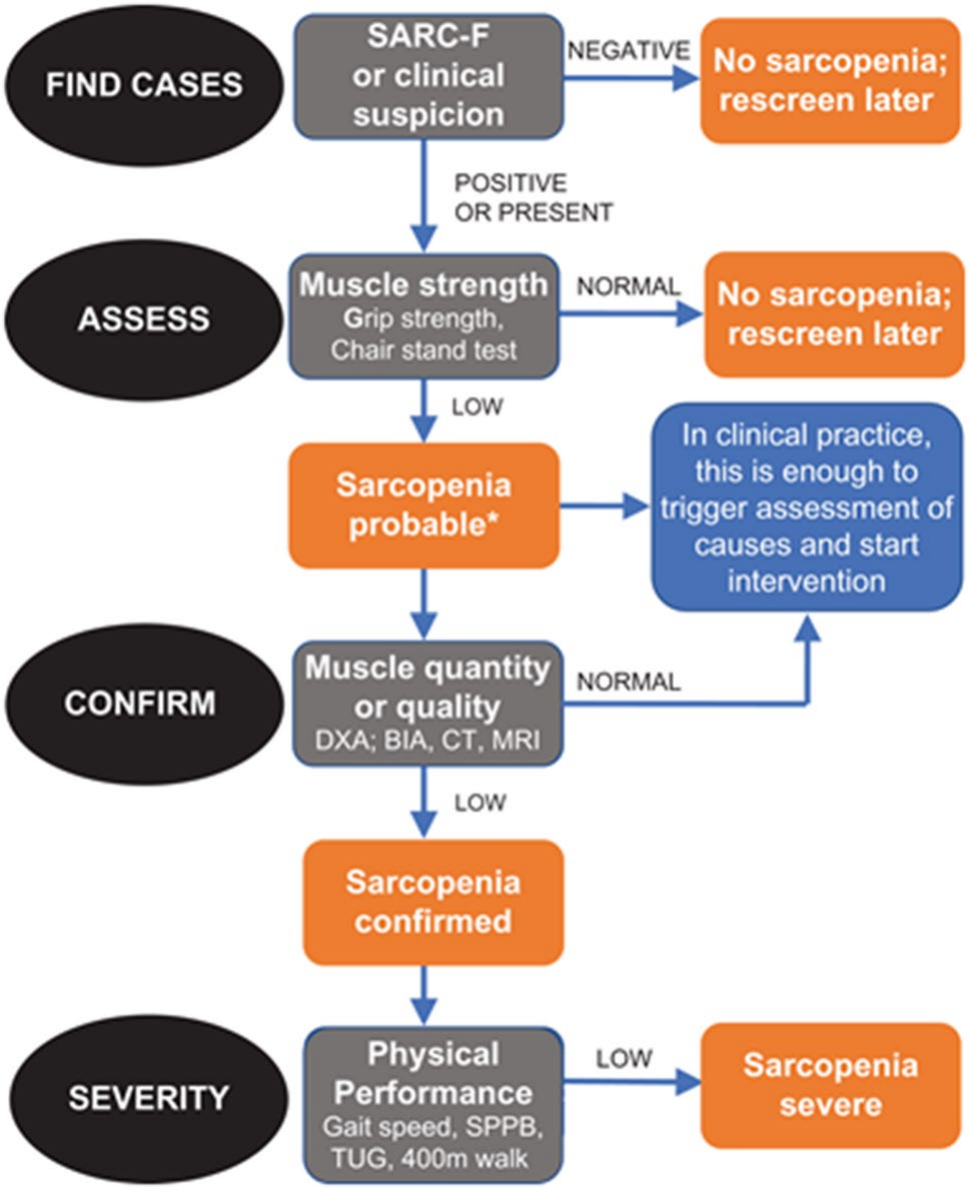
EWGSOP2 algorithm for case-finding and diagnosis of sarcopenia, and determination of severity [111]. *BIA* bioimpedance analysis, *CT* computed tomography, *DXA* dual-energy X-ray absorptiometry, *MRI* magnetic resonance imaging, *SPPB* short physical performance battery, *TUG* timed up-and-go test. Reprinted from: Cruz-Jentoft AJ et al. [111] with permission from Oxford University Press 2019

The revised European consensus on the definition and diagnosis of sarcopenia (EWGSOP2) recognizes that strength is better than muscle mass in predicting adverse outcomes [111, 119]. Thus, low muscle strength is the primary parameter of sarcopenia, and the diagnosis is confirmed by the presence of low muscle quantity or quality; when low muscle strength, low muscle quality/quantity and low physical performance are all detected sarcopenia is considered as severe [111]. The EWGSOP2 recommends an algorithm for case-finding, diagnosis and severity determination for identification of people with sarcopenia, or at risk (Fig. 5). The EWGSOP2 provides cut-off points for different parameters (set at − 2.5 SD below mean reference values for healthy young European adults) (Table 2), which will help to harmonize sarcopenia studies.

**Table 2:** EWGSOP2 cut-off values for determination of sarcopenia in the European population. Table compiled from Cruz-Jentoft AJ et al. [111]

Measurement	Tools	Cut-off values	
		Men	Women
Muscle mass	Appendicular skeletal mass	< 20 kg	< 15 kg
	Appendicular skeletal mass/height^2^	< 7 kg/m^2^	< 6 kg/m^2^
Muscle strength	Handgrip strength	< 27 kg	< 16 kg
	Chair stand	> 15 s for 5 rises	
Physical performance	Gait speed	≤ 0.8 m/s	
	Short Physical Performance Battery	≤ 8-point score	
	Timed up-and-go test	≥ 20 s	
	400-m walk test	Non-completion or ≥ 6 min for completion	

### EU practice for management of musculoskeletal health

#### Resistance exercise and protein intake

The ESCEO recommends that the management of sarcopenia should primarily be patient-centered and involve the combination of both resistance and endurance-based activity programs with or without dietary interventions [120]. Prolonged resistance-type exercise training is shown to increase skeletal muscle mass and strength, augment intrinsic capacity, improve glycemia and lipidemia, and reduce blood pressure in healthy elderly men and women [121, 122].

Functional impairment occurs alongside poor nutritional status among older adults receiving home care [123]. Contractile loading of skeletal muscle, through resistive-type exercise and amino acid ingestion both act as a strong stimulus for muscle protein synthesis and, when combined, can induce a net positive protein balance and muscle hypertrophy [124, 125]. Given that muscle protein synthesis in older muscles displays a blunted response to anabolic stimuli compared with the young [126], the combined effect of contractile and nutrient interventions to optimize muscle anabolism could be important for counteracting sarcopenia [124, 127]. The European Society for Clinical Nutrition and Metabolism (ESPEN) has provided recommendations for healthcare professionals to help older adults sustain muscle strength and function into older age, in the form of practical guidance for optimal dietary protein intake and exercise for older adults (aged > 65 years) (Table 3) [128]. The timing of protein ingestion and exercise is also important to optimize muscle protein synthesis. Consuming an adequate amount of high-quality protein at each meal, in combination with physical activity, may delay the onset of sarcopenia, slow its progression, and reduce the magnitude of its functional consequences [129, 130]. The Sarcopenia and Physical fRailty IN older people: multi-componenT Treatment strategies (SPRINTT) trial is testing whether a multicomponent intervention in high-risk older persons based on long-term structured physical activity, nutritional counseling/dietary intervention, and an information and communication technology intervention will prevent mobility disability for up to 3 years [131].

**Table 3:** ESPEN guidance for optimal dietary protein intake and exercise for older adults aged > 65 years. Table compiled from Deutz NE et al. [128]

Recommendations
For healthy older adults, a diet that includes at least 1.0–1.2 g protein/kg body weight/day
For certain older adults who have acute or chronic illnesses: 1.2–1.5 g protein/kg body weight/day may be indicated, with even higher intake for individuals with severe illness or injury
Daily physical activity for all older adults, as long as activity is possible; also, resistance training when possible as part as an overall fitness regimen

A systematic review has found only a limited effect of protein, essential amino acids (EAA), β-hydroxy β-methylbutyrate (HMB), creatine, dehydroepiandrosterone (DHEA) and fatty acid supplementation on muscle mass, muscle strength and physical performance of elderly subjects, although the overall quality of the evidence was judged to be low and well-designed and appropriately powered randomized controlled trials are needed [132].

#### Calcium and vitamin D

Calcium is largely involved in skeletal muscle-regulation and maintenance; it is also key to the activation of glycolytic metabolism and mitochondrial energy metabolism [133]. The major role of vitamin D in humans is related to bone metabolism and mineral homeostasis, and there is evidence that vitamin D contributes to calcium uptake and regulation in muscle cells [134]. Thus, calcium is important not just for bone health, but also for proper function of skeletal muscle. Worldwide there is much variation in dietary calcium intake [51] and vitamin D status [52]. Calcium intake and intestinal calcium absorption decrease with aging, as does adaptation to a low calcium diet and renal retention of calcium. Vitamin D intake and endogenous production of vitamin D is also reduced with aging, and deficiencies in both dietary calcium intake and vitamin D intake and synthesis are linked to bone loss and fracture risk [135]. Successive meta-analyses conclude that vitamin D supplementation reduces the risk of falling (number of fallers) by 8–22% [133]. The evidence base supports the use of calcium in combination with vitamin D supplementation (RR 0.87; 95% CI 0.77, 0.97), rather than as the sole agent (RR 0.90; 95% CI 0.80, 1.00) for reduction of fracture risk (*N* = 52,625), but with the magnitude of effect being modest [136]. Combined vitamin D and calcium supplementation reduces fracture risk in postmenopausal women (*N* = 52,915), although the effects are smaller among community-dwelling older adults than among institutionalized elderly persons [137]. Consequently, the ESCEO-IOF recommends that calcium with concomitant vitamin D supplementation is supported for patients at high risk of calcium and vitamin D insufficiency, and in those who are receiving treatment for osteoporosis [133].

There appears to be a U-shaped association between vitamin D level and risk of falls or fracture whether analyzed by dose or serum 25-hydroxyvitamin D levels; there was no decrease in falls on low vitamin D doses (400, 800 IU), a significant decrease on medium doses (1600, 2400, 3200 IU) (*p* = 0.020) and no decrease on high doses (4000, 4800 IU) compared with placebo (*p* = 0.55) [138–141]. In a retrospective observational study (*N* = 247,574; median follow-up 3.07 years), a reverse J-shaped relation was identified between serum 25-hydroxyvitamin D level and all-cause mortality, indicating not only a lower limit but also an upper limit. The lowest mortality risk was at 50–60 nmol/L [142].

However, in a recent meta-analysis of 81 RCTs (*N* = 53,537), vitamin D had no effect on total fracture (*N* = 44,790; RR 1.00; 95% CI 0.93, 1.07), hip fracture (*N* = 36 655; RR 1.11, 95% CI 0.97, 1.26), or falls (*N* = 34,144; RR 0.97; 95% CI 0.93, 1.02). Results were similar for high-dose versus low-dose vitamin D and in subgroup analyses of trials using doses > 800 IU per day. In pooled analyses, there were no clinically relevant between-group differences in BMD at any site (range − 0.16 to 0.76% over 1–5 years) [143]. Thus, further research is needed to better ascertain the effects of daily vitamin D dosing, with or without calcium. In addition, vitamin D supplementation is shown to have a small positive impact on muscle strength, but additional studies are needed to define optimal treatment modalities, including dose, mode of administration, and duration [144].

### Prevalence of sarcopenia in China

Asia is a rapidly aging region with a huge population in which there is an urgent need for investigation and management of sarcopenia. However, understanding of sarcopenia from a healthcare point of view is lacking; the prevalence of sarcopenia and definition should be based upon clinically relevant low muscle strength and muscle mass, identifying cut-off values for incident mobility limitation. The proper selection of cut-off values of handgrip strength, walking speed, and skeletal muscle indices with full considerations of gender and ethnic differences are of critical importance in determining the prevalence of sarcopenia. Selecting appropriate sarcopenia diagnostic cut-off values for all measurements in the Asian populations is challenging. The Asian Working group for Sarcopenia (AWGS) sets out recommendations for sarcopenia screening for community-dwelling older people and cut-off values for muscle mass measurement, strength and performance (Table 4) [112, 145]. Using these cut-off values, the prevalence of sarcopenia estimated by the AWGS criteria ranges between 4.1 and 11.5% of the general older population [146]. By comparison, the prevalence of sarcopenia among older people in Taiwan ranged from 5.8 to 14.9% in men and 4.1–16.6% in women according to International Working Group on Sarcopenia (IWGS) and EWGSOP criteria [147].

**Table 4:** Measurement tools and cut-off values for determination of sarcopenia in the Asian population. Table compiled from CSOBR [145]

Measurement	Tools	Cut-off values	
		Men	Women
Muscle mass^a^	Dual-energy X-ray absorptiometry	7 kg/m^2^	5.4 kg/m^2^
	Bioimpedance analysis	7 kg/m^2^	5.7 kg/m^2^
Muscle strength	Handgrip strength	26 kg	18 kg
	Knee flexion/extension (quadriceps strength)	18 kg	16 kg
Physical performance	6-m usual gait speed	0.8 m/s	0.8 m/s

^a^Relative appendicular skeletal mass/height^2^

A cross-sectional survey was conducted in Shanghai, Eastern China, to evaluate the prevalence of loss of muscle mass corresponding to sarcopenia in Chinese men and women and compare the results with the prevalence in other populations. Class 1 and class 2 sarcopenia were defined as the appendicular lean mass (ALM) index (ALM/height^2^) 1 and 2 SD below the sex-specific means for young adults. The reference values for class 1 and 2 sarcopenia were 7.01 and 6.08 kg/m^2^ in men and 5.42 and 4.79 kg/m^2^ in women based upon muscle mass measurement by DXA. The prevalence of sarcopenia was 4.8% in women and 13.2% in men aged ≥ 70 years, which is lower than that in Caucasian populations, but similar to that in Japanese and Korean populations [148].

The prevalence of sarcopenia in an elderly suburb-dwelling Chinese population (in Tianjin) was found to be 6.4% in men and 11.5% in women aged ≥ 60 years using the AWGS criteria. Sarcopenia was inversely associated with BMI and positively associated with diabetes, peptic ulcer, and daily drinking [149]. A comparison of sarcopenia prevalence in community-dwelling elderly has found that rural elders are more vulnerable to sarcopenia than urban elders in a sample of western China’s elderly population (around Beijing). The prevalence of sarcopenia was 13.1% in rural elders and 7.0% in urban elders (aged 70.6 ± 6.7 years). Age (OR 1.22; 95% CI 1.15–1.29), female (OR 1.71; 95% CI 1.20, 5.65), malnutrition or at risk for malnutrition (OR 3.53; 95% CI 1.68, 7.41), rural residence (OR 2.15; 95% CI 1.33, 4.51), and polypharmacy (OR 1.23; 95% CI 1.06, 1.44) were independently associated with sarcopenia [150].

Among older hospitalized patients in the Chengdu region, the prevalence of sarcopenia was found to be 31% (age 81.0 ± 8.0 years). Being female (OR 4.75; 95% CI 2.45, 9.20), smoking (OR 2.94; 95% CI 1.26, 6.69), cognitive impairment (OR 2.08; 95% CI 1.10, 3.95), polypharmacy (OR 2.36; 95% CI 1.28–4.34) and BMI (OR 0.75; 95% CI 0.68, 0.83) were independently associated with sarcopenia [151]. Sarcopenia is highly prevalent among elderly Chinese nursing home residents regardless of the diagnostic criteria used (31–38%). Malnutrition was independently associated with sarcopenia; calf circumference was negatively associated with sarcopenia, as was ≥ 1 fall in the past year [152].

The decline in muscle mass, grip strength and gait speed has been examined in a longitudinal study of community-dwelling older Chinese people (> 64 years) (*N* = 3018). After 4 years, the rate of loss of muscle mass was modest (− 1.59% and − 2.02% in men and women), but the decline in gait speed was rapid (− 8.2% in men and − 9.0% in women) and the decline in grip strength was particularly fast in older Chinese women who lost 10% in 2 years (men lost 4% in 2 years) [153].

### Management of sarcopenia in China

**Fig. 6:**
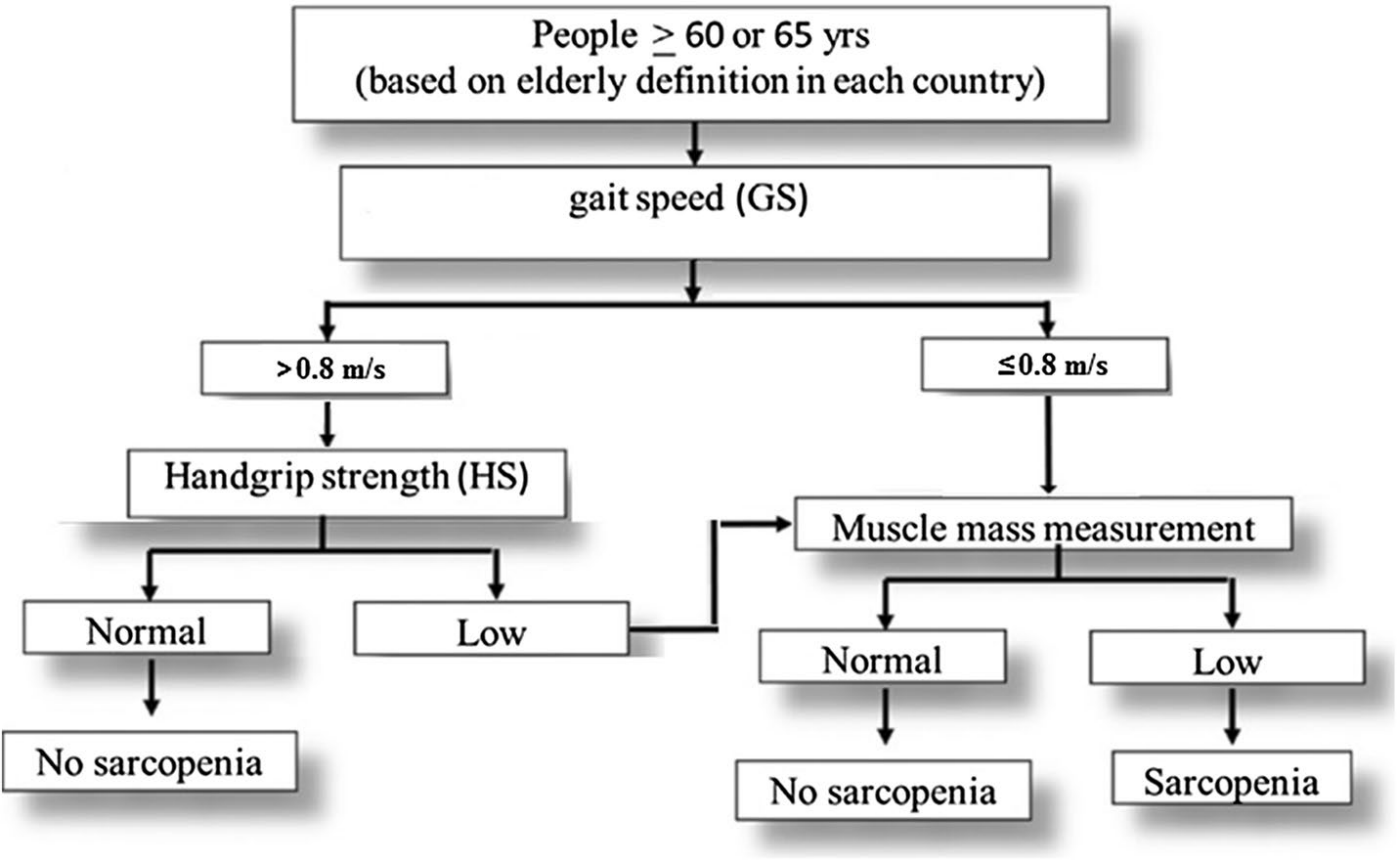
Screening and diagnosis of sarcopenia in China. Adapted from CSOBMR [145]

Several guidelines have been published by various Chinese societies to advise on the screening and diagnosis of sarcopenia (Fig. 6) [145], with management of sarcopenia in the elderly population focused on using a combination of nutritional requirements and physical exercises [145, 154–157]. Calorie intake plays an important role in maintaining muscle mass in older people, and studies show that oral nutrition supplementation besides increasing protein intake could help older people with sarcopenia. A study of ambulatory elderly people (≥ 65 years) (*N* = 74) with reduced handgrip strength and/or gait speed recruited from five hospitals in China, randomized subjects to receive nutritional supplementation (400 kcal/days), protein supplementation (20 g/days) or the control group. All subjects were given healthy lifestyle education. After 3 months, handgrip strength and gait speed in the three groups were improved compared to baseline. Caloric supplementation may have had greater effect in maintaining muscle mass than protein supplementation in the elderly, although neither produced any additional effect on muscular function as compared with healthy lifestyle education [158].

β-Hydroxy β-methylbutyrate has been shown to be effective and superior to other types of protein supplements to attenuate loss of muscle mass, strength and function; in studies including older people with sarcopenia or frailty (*N* = 203), lean body mass increased and muscle strength and function were preserved following HMB supplementation [159]. Studies of community-dwelling older people with sarcopenia have shown that HMB could play an additional role in increasing muscle mass and handgrip strength in combination with oral nutrition supplementation [160–163].

A further study is investigating the effect of nutrition plus an exercise program on physical function in sarcopenic obese elderly people [164, 165]. Resistance training, aerobic training or combination training in older obese adults with sarcopenia all demonstrate benefits in terms of increased muscle mass, reduced total fat mass, and visceral fat area after 12 weeks [166, 167]. Among elderly people with sarcopenia, participating in kettlebell training significantly increased the sarcopenia index, grip strength, back strength, and peak expiratory flow over 8 weeks, which was maintained for a further 4 weeks post-training [168]. Long-term Tai Chi exercise over 6–12 months is shown to benefit muscle strength of the lower limbs [169].

## Conclusions

This article outlines approaches to identifying and treating musculoskeletal disorders in Europe and China. There are many similarities in approach between the different regions, which provide reassurance; however, differences may exist due to cultural and regional variations in organization of patient care. Challenges exist in both regions for identification of patients with osteoporosis, and both the ESCEO-IOF and CMA guidance outline a similar case-finding strategy to stratify people into high/middle/low-risk groups and monitor and treat accordingly (Table 5). The diagnosis of osteoporosis relies on BMD measurement, for which access to DXA equipment may vary between countries and regions, and country-specific FRAX assessment. The use of BTMs for prediction of fracture risk requires further validation, but BTMs may be used to monitor treatment. The normal ranges of BTMs in different populations need to be established.

**Table 5:** Similarities between European and Chinese guidelines for the diagnosis of osteoporosis. Table compiled from ESCEO-IOF [15] and CMA [17]

	Europe ESCEO-IOF 2019 [15]	China CMA 2017 [17]
Risk assessment	Risk factors	Risk factors IOF questionnaire OSTA
Measurement	DXA-based BMD	DXA-based BMD
Diagnostic cut-off	*T* score ≤ 2.5	*T* score ≤ 2.5
Fracture risk assessment	Country-specific FRAX	Country-specific FRAX
Risk classification	High/middle/low	High/middle/low
Treatment group	High-risk patients	High-risk patients

*CMA* Chinese Medical Association of osteoporosis and bone mineral disease branch, *ESCEO-IOF* European Society for Clinical and Economic Aspects of Osteoporosis, Osteoarthritis and Musculoskeletal diseases, *IOF* International Osteoporosis Foundation, *BMD* bone mineral density, *DXA* dual-energy X-ray absorptiometry, *FRAX* fracture risk assessment tool, *OSTA* osteo self-assessment tool for Asians

Similar challenges have arisen in the identification and management of sarcopenia, for which progress has been hampered by a lack of clear consensus on the definition and diagnosis of sarcopenia as several definitions have emerged. The recent EWGSOP2 revised consensus provides cut-off values for different parameters used for diagnosis of sarcopenia, which are similar to those identified for Asian populations (Table 6). The use of appropriate population-based cut-off values will help to harmonize future sarcopenia studies.

**Table 6:** European and Chinese guidelines for screening and diagnosis of sarcopenia: population-specific cut-off values for men and women. Table compiled from EWGSOP2 [111] and CMA [145]

Factor	Measurement	Cut-off values			
		Europe EWGSOP2 [111]		China CMA [145]	
		Men	Women	Men	Women
Muscle mass	Appendicular skeletal mass/height^2^	7 kg/m^2^	6 kg/m^2^	7 kg/m^2^	5.4 kg/m^2^
Muscle strength	Handgrip strength	27 kg	16 kg	26 kg	18 kg
Physical performance	6-m usual gait speed	0.8 m/s	0.8 m/s	0.8 m/s	0.8 m/s

CMA, Chinese Medical Association of osteoporosis and bone mineral disease branch; EWGSOP, European Working Group on Sarcopenia in Older People

The challenge faced by physicians when treating osteoarthritis is finding an effective treatment for a specific patient from the breadth of available therapies, none of which provides a complete solution. The ESCEO algorithm recommends a stepwise approach to pharmacological therapy informed by available evidence for the benefits and harms of various treatments. Similarly, guidance from the Chinese Medical Association outlines a ladder of escalating and individualized treatment according to patient-specific factors. European and Chinese guideline recommendations for the use of different drug classes to treat knee OA are outlined in Table 7, and are largely complementary.

**Table 7:** Recommendations for pharmacological treatment of knee osteoarthritis: similarities between European and Chinese guidelines. Table compiled from ESCEO [58] and CMA [59]

Treatment	Europe ESCEO 2019 [58]	China CMA 2018 [59]
Glucosamine and chondroitin	Recommend use of prescription formulations only	Selective use
Oral NSAIDs	Recommend intermittent use of oral NSAIDs, and individualized treatment based on patient risk profile	Recommend using at lowest effective dose and individualized treatment based on patient risk profile
Intra-articular injection of glucocorticoid	Selective use in patients with knee effusion	Recommend use The drug should be used no more than 2–3 times a year, and the injection interval should not be shorter than 3–6 months
Intra-articular injection of hyaluronic acid	Recommend use in patients contraindicated to NSAIDs	Recommend use
Paracetamol and opioids	Use with caution due to high propensity for adverse events	Use with caution Pay attention to the adverse reactions and addiction of opioids

*CMA* Chinese Medical Association of osteoporosis and bone mineral disease branch, *ESCEO* European Society for Clinical and Economic Aspects of Osteoporosis, Osteoarthritis and Musculoskeletal diseases, *NSAID* non-steroidal anti-inflammatory drug

In conclusion, this meeting allowed experts from both regions to share their experience and recommendations for the management of three major diseases among musculoskeletal disorders. Discussing and analyzing similarities and differences in their practice should, through a mutual enrichment of knowledge, lead to better management of these diseases in order to preserve intrinsic capacity and retard age-related degradation of physical ability.

## References

[1] WHO (2015) World report on ageing and health. http://www.who.int/ageing/events/world-report-2015-launch/en/. Accessed 24 Jan 2019

[2] Marzetti E, Calvani R, Tosato M (2017). Sarcopenia: an overview. Aging Clin Exp Res.

[3] Kanis JA, Johnell O, Oden A (2000). Long-term risk of osteoporotic fracture in Malmo. Osteoporos Int.

[4] Murray CJ, Vos T, Lozano R (2012). Disability-adjusted life years (DALYs) for 291 diseases and injuries in 21 regions, 1990–2010: a systematic analysis for the global burden of disease study 2010. Lancet.

[5] Woolf AD, Pfleger B (2003). Burden of major musculoskeletal conditions. Bull World Health Organ.

[6] Vos T, Flaxman AD, Naghavi M (2012). Years lived with disability (YLDs) for 1160 sequelae of 289 diseases and injuries 1990–2010: a systematic analysis for the global burden of disease study 2010. Lancet.

[7] Cross M, Smith E, Hoy D (2014). The global burden of hip and knee osteoarthritis: estimates from the global burden of disease 2010 study. Ann Rheum Dis.

[8] Lim SS, Vos T, Flaxman AD (2012). A comparative risk assessment of burden of disease and injury attributable to 67 risk factors and risk factor clusters in 21 regions, 1990–2010: a systematic analysis for the global burden of disease study 2010. Lancet.

[9] Woolf AD, Erwin J, March L (2012). The need to address the burden of musculoskeletal conditions. Best Pract Res Clin Rheumatol.

[10] Kanis JA, McCloskey EV, Johansson H (2013). European guidance for the diagnosis and management of osteoporosis in postmenopausal women. Osteoporos Int.

[11] Hernlund E, Svedbom A, Ivergard M (2013). Osteoporosis in the European Union: medical management, epidemiology and economic burden. A report prepared in collaboration with the International Osteoporosis Foundation (IOF) and the European Federation of Pharmaceutical Industry Associations (EFPIA). Arch Osteoporos.

[12] Shepstone L, Lenaghan E, Cooper C (2018). Screening in the community to reduce fractures in older women (SCOOP): a randomised controlled trial. Lancet.

[13] Svedbom A, Hernlund E, Ivergard M (2013). Osteoporosis in the European Union: a compendium of country-specific reports. Arch Osteoporos.

[14] Kanis JA, McCloskey EV, Johansson H (2008). A reference standard for the description of osteoporosis. Bone.

[15] Kanis JA, Cooper C, Rizzoli R, Reginster JY (2019). European guidance for the diagnosis and management of osteoporosis in postmenopausal women. Osteoporos Int.

[16] Kanis JA, Oden A, Johnell O (2007). The use of clinical risk factors enhances the performance of BMD in the prediction of hip and osteoporotic fractures in men and women. Osteoporos Int.

[17] Chinese Medical Association of Osteoporosis and Bone mineral disease branch (2017). Primary osteoporosis treatment guide. Chin J Osteoporos Bone Miner Res.

[18] Liao EY, Wu XP, Deng XG (2002). Age-related bone mineral density, accumulated bone loss rate and prevalence of osteoporosis at multiple skeletal sites in chinese women. Osteoporos Int.

[19] Cui L, Chen L, Xia W (2017). Vertebral fracture in postmenopausal chinese women: a population-based study. Osteoporos Int.

[20] Xia WB, He SL, Xu L (2012). Rapidly increasing rates of hip fracture in Beijing, China. J Bone Miner Res.

[21] Qu B, Ma Y, Yan M (2014). The economic burden of fracture patients with osteoporosis in western China. Osteoporos Int.

[22] Si L, Winzenberg TM, Jiang Q (2015). Projection of osteoporosis-related fractures and costs in china: 2010–2050. Osteoporos Int.

[23] Naylor K, Eastell R (2012). Bone turnover markers: use in osteoporosis. Nat Rev Rheumatol.

[24] Vasikaran S, Cooper C, Eastell R (2011). International Osteoporosis Foundation and International Federation of Clinical Chemistry and Laboratory Medicine position on bone marker standards in osteoporosis. Clin Chem Lab Med.

[25] Vasikaran S, Eastell R, Bruyere O (2011). Markers of bone turnover for the prediction of fracture risk and monitoring of osteoporosis treatment: a need for international reference standards. Osteoporos Int.

[26] Johansson H, Oden A, Kanis JA (2014). A meta-analysis of reference markers of bone turnover for prediction of fracture. Calcif Tissue Int.

[27] Diez-Perez A, Naylor KE, Abrahamsen B (2017). International Osteoporosis Foundation and European Calcified Tissue Society Working Group. Recommendations for the screening of adherence to oral bisphosphonates. Osteoporos Int.

[28] Devogelaer JP, Boutsen Y, Gruson D (2011). Is there a place for bone turnover markers in the assessment of osteoporosis and its treatment?. Rheum Dis Clin N Am.

[29] Jorgensen NR, Mollehave LT, Hansen YBL (2017). Comparison of two automated assays of btm (ctx and p1np) and reference intervals in a Danish population. Osteoporos Int.

[30] Eastell R, Szulc P (2017). Use of bone turnover markers in postmenopausal osteoporosis. Lancet Diabetes Endocrinol.

[31] Szulc P, Naylor K, Hoyle NR (2017). Use of CTX-I and PINP as bone turnover markers: national bone health alliance recommendations to standardize sample handling and patient preparation to reduce pre-analytical variability. Osteoporos Int.

[32] Nishizawa Y, Ohta H, Miura M (2013). Guidelines for the use of bone metabolic markers in the diagnosis and treatment of osteoporosis (2012 edition). J Bone Miner Metab.

[33] CSOBMR (2015). Guidelines from the Chinese Society of Osteoporosis and Bone Mineral Research on bone turnover markers Chin J Osteoporosis Bone Miner Res.

[34] CSOBMR (2017). Guideline of diagnosis and treatment of primary osteoporosis. Chin J Osteoporosis Bone Miner Res.

[35] Li M, Li Y, Deng W (2014). Chinese bone turnover marker study: reference ranges for c-terminal telopeptide of type I collagen and procollagen I n-terminal peptide by age and gender. PLoS One.

[36] Li M, Lv F, Zhang Z (2016). Establishment of a normal reference value of parathyroid hormone in a large healthy chinese population and evaluation of its relation to bone turnover and bone mineral density. Osteoporos Int.

[37] Zhou X-W, Wu X-Y, Luo L (2011). The relationship between bone turnover markers and bmd decreasing rates in chinese middle-aged women. Clin Chim Acta.

[38] Dai Z, Wang R, Ang LW (2016). Bone turnover biomarkers and risk of osteoporotic hip fracture in an asian population. Bone.

[39] Zhao J, Xia W, Nie M (2011). The levels of bone turnover markers in Chinese postmenopausal women: peking vertebral fracture study. Menopause.

[40] Jiajue R, Jiang Y, Wang O (2014). Suppressed bone turnover was associated with increased osteoporotic fracture risks in non-obese postmenopausal Chinese women with type 2 diabetes mellitus. Osteoporos Int.

[41] Wang J, Yan D, Hou X (2017). Association of adiposity indices with bone density and bone turnover in the Chinese population. Osteoporos Int.

[42] Liang BC, Shi ZY, Wang B (2017). Intravenous zoledronic acid 5 mg on bone turnover markers and bone mineral density in east China subjects with newly diagnosed osteoporosis: a 24-month clinical study. Orthop Surg.

[43] Li M, Zhang Z-l, Liao E-Y (2013). Effect of low-dose alendronate treatment on bone mineral density and bone turnover markers in Chinese postmenopausal women with osteopenia and osteoporosis. Menopause.

[44] Chen JF, Yang KH, Zhang ZL (2015). A systematic review on the use of daily subcutaneous administration of teriparatide for treatment of patients with osteoporosis at high risk for fracture in Asia. Osteoporos Int.

[45] Botella S, Restituto P, Monreal I (2013). Traditional and novel bone remodeling markers in premenopausal and postmenopausal women. J Clin Endocrinol Metab.

[46] Chapurlat RD, Confavreux CB (2016). Novel biological markers of bone: from bone metabolism to bone physiology. Rheumatology (Oxford).

[47] Kanis JA, Cooper C, Rizzoli R (2019). Executive summary of European guidance for the diagnosis and management of osteoporosis in postmenopausal women. Aging Clin Exp Res.

[48] Kanis JA, McCloskey EV, Johansson H, Strom O, Borgstrom F, Oden A (2008). Case finding for the management of osteoporosis with frax—assessment and intervention thresholds for the UK. Osteoporos Int.

[49] Wang O, Hu Y, Gong S (2015). A survey of outcomes and management of patients post fragility fractures in China. Osteoporos Int.

[50] Man Y, Pan W, Lu J (2016). Treatment and management of osteoporotic fractures: a nation-wide survey of 484 senior orthopaedists in China. Orthop Surg.

[51] Balk EM, Adam GP, Langberg VN (2017). Global dietary calcium intake among adults: a systematic review. Osteoporos Int.

[52] Wahl DA, Cooper C, Ebeling PR (2012). A global representation of vitamin D status in healthy populations. Arch Osteoporos.

[53] Liao RX, Yu M, Jiang Y (2014). Management of osteoporosis with calcitriol in elderly Chinese patients: a systematic review. Clin Interv Aging.

[54] Zhang ZL, Liao EY, Xia WB, Lin H, Cheng Q, Wang L (2015). Alendronate sodium/vitamin D3 combination tablet versus calcitriol for osteoporosis in Chinese postmenopausal women: a 6-month, randomized, open-label, active-comparator-controlled study with a 6-month extension. Osteoporos Int.

[55] Liao EY, Zhang ZL, Xia WB (2018). Calcifediol (25-hydroxyvitamin D) improvement and calcium-phosphate metabolism of alendronate sodium/vitamin D3 combination in Chinese women with postmenopausal osteoporosis: a post hoc efficacy analysis and safety reappraisal. BMC Musculoskelet Disord.

[56] Liao EY, Zhang ZL, Xia WB (2018). Clinical characteristics associated with bone mineral density improvement after 1-year alendronate/vitamin D3 or calcitriol treatment: exploratory results from a phase 3, randomized, controlled trial on postmenopausal osteoporotic women in China. Medicine (Baltimore).

[57] Bruyere O, Cooper C, Pelletier JP (2014). An algorithm recommendation for the management of knee osteoarthritis in Europe and internationally: a report from a task force of the European Society for Clinical and Economic Aspects of Osteoporosis and Osteoarthritis (ESCEO). Semin Arthritis Rheum.

[58] Bruyere O, Honvo G, Veronese N (2019). An updated algorithm recommendation for the management of knee osteoarthritis from the European Society for clinical and economic aspects of osteoporosis, osteoarthritis and musculoskeletal diseases (ESCEO). Semin Arthritis Rheum.

[59] Xing-Ming S (2018). Guidelines for the diagnosis and treatment of osteoarthritis in China (2018). Chin J Orthop.

[60] Zhang Z, Duan X, Gu J (2016). The European Society for Clinical and Economic Aspects of Osteoporosis and Osteoarthritis (ESCEO) algorithm for the management of knee osteoarthritis is applicable to chinese clinical practice: a consensus statement of leading Chinese and ESCEO osteoarthritis experts. Chin J Pract Internal Med.

[61] Honvo G, Reginster J-Y, Rabenda V (2019). Safety of symptomatic slow-acting drugs for osteoarthritis: outcomes of a systematic review and meta-analysis. Drugs Aging.

[62] Bruyere O, Cooper C, Al-Daghri NM (2017). Inappropriate claims from non-equivalent medications in osteoarthritis: a position paper endorsed by the European Society for Clinical and Economic Aspects of Osteoporosis, Osteoarthritis and Musculoskeletal Diseases (ESCEO). Aging Clin Exp Res.

[63] Derry S, Conaghan P, Da Silva JA (2016). Topical NSAIDs for chronic musculoskeletal pain in adults. Cochrane Database Syst Rev.

[64] Rannou F, Pelletier JP, Martel-Pelletier J (2016). Efficacy and safety of topical NSAIDs in the management of osteoarthritis: evidence from real-life setting trials and surveys. Semin Arthritis Rheum.

[65] Honvo G, Leclercq V, Geerinck A (2019). Safety of topical non-steroidal anti-inflammatory drugs in osteoarthritis: outcomes of a systematic review and meta-analysis. Drugs Aging.

[66] Curtis E, Fuggle N, Shaw S (2019). Safety of cyclo-oxygenase-2 inhibitors in osteoarthritis: outcomes of a systematic review and meta-analysis. Drugs Aging.

[67] Cooper C, Chapurlat R, Al-Daghri N (2019). Safety of oral non-selective non-steroidal anti-inflammatory drugs in osteoarthritis: what does the literature say?. Drugs Aging.

[68] Bellamy N, Campbell J, Robinson V (2006). Viscosupplementation for the treatment of osteoarthritis of the knee. Cochrane Database Syst Rev.

[69] Bannuru RR, Vaysbrot EE, Sullivan MC (2014). Relative efficacy of hyaluronic acid in comparison with nsaids for knee osteoarthritis: a systematic review and meta-analysis. Semin Arthritis Rheum.

[70] Maheu E, Rannou F, Reginster JY (2016). Efficacy and safety of hyaluronic acid in the management of osteoarthritis: evidence from real-life setting trials and surveys. Semin Arthritis Rheum.

[71] Maheu E, Bannuru RR, Herrero-Beaumont G (2019). Why we should definitely include intra-articular hyaluronic acid as a therapeutic option in the management of knee osteoarthritis: results of an extensive critical literature review. Semin Arthritis Rheum.

[72] Honvo G, Reginster JY, Rannou F (2019). Safety of intra-articular hyaluronic acid injections in osteoarthritis: outcomes of a systematic review and meta-analysis. Drugs Aging.

[73] Bannuru RR, Natov NS, Obadan IE (2009). Therapeutic trajectory of hyaluronic acid versus corticosteroids in the treatment of knee osteoarthritis: a systematic review and meta-analysis. Arthritis Rheum.

[74] McAlindon TE, LaValley MP, Harvey WF (2017). Effect of intra-articular triamcinolone vs saline on knee cartilage volume and pain in patients with knee osteoarthritis: a randomized clinical trial. JAMA.

[75] Cepeda MS, Camargo F, Zea C (2006). Tramadol for osteoarthritis. Cochrane Database Syst Rev.

[76] da Costa BR, Nuesch E, Kasteler R (2014). Oral or transdermal opioids for osteoarthritis of the knee or hip. Cochrane Database Syst Rev.

[77] Fuggle N, Curtis E, Shaw S (2019). Safety of opioids in osteoarthritis: outcomes of a systematic review and meta-analysis. Drugs Aging.

[78] Conaghan PG, Arden N, Avouac B (2019). Safety of paracetamol in osteoarthritis: what does the literature say?. Drugs Aging.

[79] Roberts E, Delgado Nunes V, Buckner S (2016). Paracetamol: not as safe as we thought? A systematic literature review of observational studies. Ann Rheum Dis.

[80] Machado GC, Maher CG, Ferreira PH (2015). Efficacy and safety of paracetamol for spinal pain and osteoarthritis: systematic review and meta-analysis of randomised placebo controlled trials. BMJ.

[81] Curhan GC, Knight EL, Rosner B (2004). Lifetime nonnarcotic analgesic use and decline in renal function in women. Arch Intern Med.

[82] Kurth T, Glynn RJ, Walker AM (2003). Analgesic use and change in kidney function in apparently healthy men. Am J Kidney Dis.

[83] de Vries F, Setakis E, van Staa TP (2010). Concomitant use of ibuprofen and paracetamol and the risk of major clinical safety outcomes. Br J Clin Pharmacol.

[84] Chan AT, Manson JE, Albert CM (2006). Nonsteroidal antiinflammatory drugs, acetaminophen, and the risk of cardiovascular events. Circulation.

[85] Lipworth L, Friis S, Mellemkjaer L (2003). A population-based cohort study of mortality among adults prescribed paracetamol in denmark. J Clin Epidemiol.

[86] Bhala N, Emberson J, Merhi A, Abramson S, Arber N, Coxib and traditional NSAID Trialists’ (CNT) Collaboration (2013). Vascular and upper gastrointestinal effects of non-steroidal anti-inflammatory drugs: meta-analyses of individual participant data from randomised trials. Lancet.

[87] Wang X, Tian HJ, Yang HK (2011). Meta-analysis: cyclooxygenase-2 inhibitors are no better than nonselective nonsteroidal anti-inflammatory drugs with proton pump inhibitors in regard to gastrointestinal adverse events in osteoarthritis and rheumatoid arthritis. Eur J Gastroenterol Hepatol.

[88] Bally M, Beauchamp ME, Abrahamowicz M (2018). Risk of acute myocardial infarction with real-world nsaids depends on dose and timing of exposure. Pharmacoepidemiol Drug Saf.

[89] Gunter BR, Butler KA, Wallace RL (2017). Non-steroidal anti-inflammatory drug-induced cardiovascular adverse events: a meta-analysis. J Clin Pharm Ther.

[90] Nissen SE, Yeomans ND, Solomon DH (2016). Cardiovascular safety of celecoxib, naproxen, or ibuprofen for arthritis. N Engl J Med.

[91] Ungprasert P, Srivali N, Thongprayoon C (2016). Nonsteroidal anti-inflammatory drugs and risk of incident heart failure: a systematic review and meta-analysis of observational studies. Clin Cardiol.

[92] Liu Q, Wang S, Lin J (2018). The burden for knee osteoarthritis among chinese elderly: estimates from a nationally representative study. Osteoarthr Cartil.

[93] Xing Dan LJ (2018). Tracing the scientific outputs in the field of osteoarthritis study in china based on publications in the web of science (2013-2017). Chin J Orthop.

[94] Li Y, Su Y, Chen S (2016). The effects of resistance exercise in patients with knee osteoarthritis: a systematic review and meta-analysis. Clin Rehabil.

[95] Fangzhou HZW (2018). Efficacy and gastrointestinal adverse reaction of non-steroidal anti-inflammatory drugs in chinese patients with osteoarthritis: network meta-analysis. Chin J Jt Surg (Electron Ed).

[96] Liu A (2016). Sodium hyaluronate versus physiological saline injection for knee osteoarthritis: a meta-analysis. Orthoped J China.

[97] Yu W, Xu P, Huang G (2018). Clinical therapy of hyaluronic acid combined with platelet-rich plasma for the treatment of knee osteoarthritis. Exp Ther Med.

[98] Lin K-Y, Yang C-C, Hsu C-J (2019). Intra-articular injection of platelet-rich plasma is superior to hyaluronic acid or saline solution in the treatment of mild to moderate knee osteoarthritis: a randomized, double-blind, triple-parallel, placebo-controlled clinical trial. Arthroscopy.

[99] Jiangang CAO (2015). The clinical research of chinese traditional medicine jintiange capsule for the treatment of osteoarthritis. Chin J Osteoporos.

[100] Bian W, Liu F, Tang X (2018). Comparison study of unicompartmental and total knee arthroplasties in treatment of unicompartmental osteoarthritis. Chin J Jt Surg (Electron Ed).

[101] Lu J, Tang S, Wang Y (2018). Clinical outcomes of closing- and opening-wedge high tibial osteotomy for treatment of anteromedial unicompartmental knee osteoarthritis. J Knee Surg (EFirst).

[102] Su X, Li C, Liao W (2018). Comparison of arthroscopic and conservative treatments for knee osteoarthritis: A 5-year retrospective comparative study. Arthrosc J Arthrosc Relat Surg.

[103] Xu T, Lao Y, Wang J (2017). Mid-term results of oxford phase-3 medial unicompartmental knee arthroplasty for medial arthritis in chinese patients. ANZ J Surg.

[104] Xie L, Tintani F, Wang X (2016). Systemic neutralization of tgf-beta attenuates osteoarthritis. Ann N Y Acad Sci.

[105] Lu J, Zhang H, Cai D (2018). Positive-feedback regulation of subchondral h-type vessel formation by chondrocyte promotes osteoarthritis development in mice. J Bone Miner Res.

[106] Li H, Wang D, Yuan Y (2017). New insights on the mmp-13 regulatory network in the pathogenesis of early osteoarthritis. Arthritis Res Ther.

[107] Roemer FW, Eckstein F, Hayashi D (2014). The role of imaging in osteoarthritis. Best Pract Res Clin Rheumatol.

[108] Peterfy CG, Schneider E, Nevitt M (2008). The osteoarthritis initiative: report on the design rationale for the magnetic resonance imaging protocol for the knee. Osteoarthr Cartil.

[109] Guermazi A, Roemer FW, Haugen IK (2013). MRI-based semiquantitative scoring of joint pathology in osteoarthritis. Nat Rev Rheumatol.

[110] Cruz-Jentoft AJ, Baeyens JP, Bauer JM (2010). Sarcopenia: European consensus on definition and diagnosis: report of the European Working Group on Sarcopenia in Older People. Age Ageing.

[111] Cruz-Jentoft AJ, Bahat G, Bauer J (2019). Sarcopenia: revised European consensus on definition and diagnosis. Age Ageing.

[112] Chen LK, Liu LK, Woo J (2014). Sarcopenia in Asia: consensus report of the Asian working group for sarcopenia. J Am Med Dir Assoc.

[113] Fielding RA, Vellas B, Evans WJ (2011). Sarcopenia: an undiagnosed condition in older adults. Current consensus definition: prevalence, etiology, and consequences. International Working Group on Sarcopenia. J Am Med Dir Assoc.

[114] Studenski SA, Peters KW, Alley DE (2014). The FNIH sarcopenia project: rationale, study description, conference recommendations, and final estimates. J Gerontol A Biol Sci Med Sci.

[115] Morley JE, Abbatecola AM, Argiles JM (2011). Sarcopenia with limited mobility: an international consensus. J Am Med Dir Assoc.

[116] Locquet M, Beaudart C, Reginster JY (2018). Comparison of the performance of five screening methods for sarcopenia. Clin Epidemiol.

[117] Bischoff-Ferrari HA, Orav JE, Kanis JA (2015). Comparative performance of current definitions of sarcopenia against the prospective incidence of falls among community-dwelling seniors age 65 and older. Osteoporos Int.

[118] Beaudart C, Locquet M, Reginster JY (2018). Quality of life in sarcopenia measured with the SARQOL: impact of the use of different diagnosis definitions. Aging Clin Exp Res.

[119] Locquet M, Beaudart C, Petermans J (2019). EWGSOP2 versus EWGSOP1: Impact on the prevalence of sarcopenia and its major health consequences. J Am Med Dir Assoc.

[120] Beaudart C, McCloskey E, Bruyere O (2016). Sarcopenia in daily practice: assessment and management. BMC Geriatr.

[121] Leenders M, Verdijk LB, Van der Hoeven L (2013). Protein supplementation during resistance-type exercise training in the elderly. Med Sci Sports Exerc.

[122] Straight CR, Lindheimer JB, Brady AO (2016). Effects of resistance training on lower-extremity muscle power in middle-aged and older adults: a systematic review and meta-analysis of randomized controlled trials. Sports Med.

[123] Kiesswetter E, Pohlhausen S, Uhlig K (2013). Malnutrition is related to functional impairment in older adults receiving home care. J Nutr Health Aging.

[124] Breen L, Phillips SM (2013). Interactions between exercise and nutrition to prevent muscle waste during ageing. Br J Clin Pharmacol.

[125] Cermak NM, Res PT, de Groot LC (2012). Protein supplementation augments the adaptive response of skeletal muscle to resistance-type exercise training: a meta-analysis. Am J Clin Nutr.

[126] Wall BT, Gorissen SH, Pennings B (2015). Aging is accompanied by a blunted muscle protein synthetic response to protein ingestion. PLoS ONE.

[127] Calvani R, Miccheli A, Landi F (2013). Current nutritional recommendations and novel dietary strategies to manage sarcopenia. J Frailty Aging.

[128] Deutz NE, Bauer JM, Barazzoni R (2014). Protein intake and exercise for optimal muscle function with aging: recommendations from the ESPEN expert group. Clin Nutr.

[129] Paddon-Jones D, Campbell WW, Jacques PF (2015). Protein and healthy aging. Am J Clin Nutr.

[130] Marzetti E, Calvani R, Tosato M (2017). Physical activity and exercise as countermeasures to physical frailty and sarcopenia. Aging Clin Exp Res.

[131] Landi F, Cesari M, Calvani R (2017). The “sarcopenia and physical frailty in older people: multi-component treatment strategies” (SPRINTT) randomized controlled trial: design and methods. Aging Clin Exp Res.

[132] Beaudart C, Rabenda V, Simmons M (2018). Effects of protein, essential amino acids, b-hydroxy b-methylbutyrate, creatine, dehydroepiandrosterone and fatty acid supplementation on muscle mass, muscle strength and physical performance in older people aged 60 years and over. A systematic review on the literature. J Nutr Health Aging.

[133] Harvey NC, Biver E, Kaufman JM (2017). The role of calcium supplementation in healthy musculoskeletal ageing: an expert consensus meeting of the European Society for Clinical and Economic Aspects of Osteoporosis, Osteoarthritis and Musculoskeletal Diseases (ESCEO) and the International Foundation for Osteoporosis (IOF). Osteoporos Int.

[134] Carmeliet G, Verstuyf A, Maes C, Seibel MJ, Robins SP, Bilezikian JP (2006). Chapter 18—the vitamin D hormone and its nuclear receptor: Mechanisms involved in bone biology. Dynamics of bone and cartilage metabolism.

[135] Rizzoli R, Bonjour J-P, Seibel MJ, Robins SP, Bilezikian JP (2006). Chapter 20—physiology of calcium and phosphate homeostases. Dynamics of bone and cartilage metabolism.

[136] Tang BM, Eslick GD, Nowson C (2007). Use of calcium or calcium in combination with vitamin D supplementation to prevent fractures and bone loss in people aged 50 years and older: a meta-analysis. Lancet.

[137] Chung M, Lee J, Terasawa T (2011). Vitamin D with or without calcium supplementation for prevention of cancer and fractures: an updated meta-analysis for the US preventive services task force. Ann Intern Med.

[138] Bleicher K, Cumming RG, Naganathan V (2014). U-shaped association between serum 25-hydroxyvitamin D and fracture risk in older men: results from the prospective population-based champ study. J Bone Miner Res.

[139] Khaw KT, Stewart AW, Waayer D (2017). Effect of monthly high-dose vitamin D supplementation on falls and non-vertebral fractures: secondary and post hoc outcomes from the randomised, double-blind, placebo-controlled VIDA trial. Lancet Diabetes Endocrinol.

[140] Smith LM, Gallagher JC, Suiter C (2017). Medium doses of daily vitamin D decrease falls and higher doses of daily vitamin D3 increase falls: a randomized clinical trial. J Steroid Biochem Mol Biol.

[141] Sanders KM, Stuart AL, Williamson EJ (2010). Annual high-dose oral vitamin D and falls and fractures in older women: a randomized controlled trial. JAMA.

[142] Durup D, Jorgensen HL, Christensen J (2012). A reverse J-shaped association of all-cause mortality with serum 25-hydroxyvitamin D in general practice: the COPD study. J Clin Endocrinol Metab.

[143] Bolland MJ, Grey A, Avenell A (2018). Effects of vitamin D supplementation on musculoskeletal health: a systematic review, meta-analysis, and trial sequential analysis. Lancet Diabetes Endocrinol.

[144] Beaudart C, Buckinx F, Rabenda V (2014). The effects of vitamin d on skeletal muscle strength, muscle mass, and muscle power: a systematic review and meta-analysis of randomized controlled trials. J Clin Endocrinol Metab.

[145] Chinese Medical Association of Osteoporosis and Bone mineral disease branch (2016). Expert consensus on sarcopenia. Chin J Osteoporos Bone Miner Res.

[146] Chen LK, Lee WJ, Peng LN (2016). Recent advances in sarcopenia research in Asia: 2016 update from the asian working group for sarcopenia. J Am Med Dir Assoc.

[147] Lee WJ, Liu LK, Peng LN (2013). Comparisons of sarcopenia defined by IWGS and EWGSOP criteria among older people: results from the i-lan longitudinal aging study. J Am Med Dir Assoc.

[148] Cheng Q, Zhu X, Zhang X (2014). A cross-sectional study of loss of muscle mass corresponding to sarcopenia in healthy chinese men and women: reference values, prevalence, and association with bone mass. J Bone Miner Metab.

[149] Han P, Kang L, Guo Q (2016). Prevalence and factors associated with sarcopenia in suburb-dwelling older Chinese using the Asian working group for sarcopenia definition. J Gerontol A Biol Sci Med Sci.

[150] Gao L, Jiang J, Yang M (2015). Prevalence of sarcopenia and associated factors in chinese community-dwelling elderly: comparison between rural and urban areas. J Am Med Dir Assoc.

[151] Hao Q, Hu X, Xie L (2018). Prevalence of sarcopenia and associated factors in hospitalised older patients: a cross-sectional study. Australas J Ageing.

[152] Zeng Y, Hu X, Xie L (2018). The prevalence of sarcopenia in Chinese elderly nursing home residents: a comparison of 4 diagnostic criteria. J Am Med Dir Assoc.

[153] Auyeung TW, Lee SW, Leung J (2014). Age-associated decline of muscle mass, grip strength and gait speed: a 4-year longitudinal study of 3018 community-dwelling older chinese. Geriatr Gerontol Int.

[154] Geriatrics Society of Chinese Medical Association (2017). Expert consensus on the management of sarcopenia in China. Chin J Geriatr.

[155] Chinese Medical Association (2018). Expert consensus of the intervention of sarcopenia in China. Health Guide.

[156] Chinese Society of Nutrition (2015). Expert consensus of the nutrition and exercise intervention of sarcopenia in Chinese. Acta Nutr Sinica.

[157] Chinese Nutrition Association (2015). Guidelines for nutritional treatment of sarcopenia in China. Electron J Metab Nutr Cancer.

[158] Wang Q, Liu X, Zhang P (2016). Effects of enteral nutrition and lactalbumin on muscle mass and function in the elderly. Chin J Geriatr.

[159] Oktaviana J, Zanker J, Vogrin S (2018). The effect of β-hydroxy-β-methylbutyrate (HMB) on sarcopenia and functional frailty in older persons: a systematic review. J Nutr Health Aging.

[160] Berton L, Bano G, Carraro S (2015). Effect of oral beta-hydroxy-beta-methylbutyrate (HMB) supplementation on physical performance in healthy old women over 65 years: an open label randomized controlled trial. PLoS One.

[161] Flakoll P, Sharp R, Baier S (2004). Effect of beta-hydroxy-beta-methylbutyrate, arginine, and lysine supplementation on strength, functionality, body composition, and protein metabolism in elderly women. Nutrition.

[162] Baier S, Johannsen D, Abumrad N (2009). Year-long changes in protein metabolism in elderly men and women supplemented with a nutrition cocktail of beta-hydroxy-beta-methylbutyrate (HMB), l-arginine, and l-lysine. JPEN J Parenter Enteral Nutr.

[163] De Luis DA, Izaola O, Bachiller P (2015). Effect on quality of life and handgrip strength by dynamometry of an enteral specific supplements with beta-hydroxy-beta-methylbutyrate and vitamin d in elderly patients. Nutr Hosp.

[164] Shen SS, Chu JJ, Cheng L (2016). Effects of a nutrition plus exercise programme on physical function in sarcopenic obese elderly people: study protocol for a randomised controlled trial. BMJ Open.

[165] Denison HJ, Cooper C, Sayer AA (2015). Prevention and optimal management of sarcopenia: a review of combined exercise and nutrition interventions to improve muscle outcomes in older people. Clin Interv Aging.

[166] Liao CD, Tsauo JY, Lin LF (2017). Effects of elastic resistance exercise on body composition and physical capacity in older women with sarcopenic obesity: a consort-compliant prospective randomized controlled trial. Medicine (Baltimore).

[167] Chen HT, Chung YC, Chen YJ (2017). Effects of different types of exercise on body composition, muscle strength, and IGF-1 in the elderly with sarcopenic obesity. J Am Geriatr Soc.

[168] Chen H-T, Wu H-J, Chen Y-J (2018). Effects of 8-week kettlebell training on body composition, muscle strength, pulmonary function, and chronic low-grade inflammation in elderly women with sarcopenia. Exp Gerontol.

[169] Zhou M, Peng N, Dai Q (2016). Effect of tai chi on muscle strength of the lower extremities in the elderly. Chin J Integr Med.

